# Markers of Immune Activation and Inflammation, and Non-Hodgkin Lymphoma: A Meta-Analysis of Prospective Studies

**DOI:** 10.1093/jncics/pky082

**Published:** 2019-03-05

**Authors:** Solomon B Makgoeng, Rachel S Bolanos, Christie Y Jeon, Robert E Weiss, Onyebuchi A Arah, Elizabeth C Breen, Otoniel Martínez-Maza, Shehnaz K Hussain

**Affiliations:** 1 Department of Epidemiology, Fielding School of Public Health, University of California, Los Angeles (UCLA), CA; 2 Department of Medicine, Samuel Oschin Comprehensive Cancer Institute, Cedars-Sinai Medical Center, Los Angeles, CA; 3 Departments of Obstetrics and Gynecology and Microbiology, Immunology and Molecular Genetics, David Geffen School of Medicine at UCLA, Los Angeles, CA; 4 Department of Biostatistics, Fielding School of Public Health, University of California, Los Angeles, CA; 5 Cousins Center for Psychoneuroimmunology, Jane and Terry Semel Institute for Neuroscience and Human Behavior, Department of Psychiatry and Biobehavioral Sciences, David Geffen School of Medicine, University of California, Los Angeles

## Abstract

**Background:**

Chronic inflammation and immune activation are reported to play a key role in the etiology of non-Hodgkin lymphoma (NHL). We conducted a meta-analysis on the associations between prediagnosis circulating levels of immune stimulatory markers, interleukin 6 (IL-6), IL-10, tumor necrosis factor α (TNF-α), CXCL13, soluble CD23 (sCD23), sCD27, sCD30, and the risk of NHL.

**Methods:**

Relevant studies were identified from PubMed, EMBASE, and Web of Science up to January 1, 2017. We calculated summary odds ratio (OR) estimates for the association between one natural log increase in concentration of each biomarker and NHL using random-effects models for NHL as a composite outcome and for several histological subtypes of NHL.

**Results:**

Seventeen nested case control studies were included. Elevated levels of several biomarkers were more strongly associated with increased odds of NHL: TNF-α, OR = 1.18 (95% confidence interval [CI] = 1.04 to 1.34); CXCL13, OR = 1.47 (95% CI = 1.03 to 2.08); sCD23, OR = 1.57 (95% CI = 1.21 to 2.05); sCD27, OR = 2.18 (95% CI = 1.20 to 3.98); sCD30, OR = 1.65 (95% CI = 1.22 to 2.22). In stratified analyses, IL-6, TNF-α, sCD27, and sCD30 were more strongly associated with NHL in HIV-infected individuals compared to HIV-uninfected individuals. Between-study heterogeneity was observed across multiple biomarkers for overall NHL and by subtypes.

**Conclusion:**

This meta-analysis provides evidence that elevated circulating levels of TNF-α, CXCL13, sCD23, sCD27, and sCD30 are consistently associated with an increased risk of NHL, suggesting the potential utility of these biomarkers in population risk stratification and prediction.

Profound immune dysregulation, particularly in the setting of HIV infection or solid organ transplantation, is among the strongest risk factors for non-Hodgkin lymphoma (NHL) (1). Among HIV-infected individuals, two pathogenic mechanisms have been hypothesized to contribute to AIDS-NHL (2–4). The first is the dysregulated proliferation of Epstein-Barr virus (EBV)-transformed B-cells, resulting from impairment of T-cell-mediated immunity (4). The other is chronic B-cell activation and resultant downstream processes that promote oncogenic mutations and translocations (3). In the setting of solid organ transplantation, a large fraction of NHL is attributed to EBV; however, NHL occurrence in long-term transplant survivors appears to be caused by factors other than EBV (5–7).

Less severe immune dysregulation, in the form of autoimmune conditions and subclinical immune deficiency, has been associated with increased NHL risk (1). Importantly, observational studies assessing associations between NHL and serologic measurements of immune markers, such as cytokines, chemokines, and soluble receptors, have provided evidence implicating alteration in these biomarkers in lymphomagenesis (8–11).

Two narrative reviews have been published that descriptively summarize much of the relevant literature regarding biomarkers for NHL development (3,12), but neither quantified the associations of immunological markers and NHL. A recent meta-analysis of associations between NHL and both soluble CD27 (sCD27) and sCD30 has been published (13). In this study, we aim to synthesize evidence that has accumulated in the literature (3,12,13) to quantify associations of prediagnosis biomarkers of inflammation and immune activation with subsequent NHL for a select set of biomarkers. We selected immune biomarkers included in prior reviews (3,12,13), which we hypothesize are biologically relevant to NHL etiology (interleukin [IL]-6, IL-10, CXCL13, sCD23, sCD27, sCD30, tumor necrosis factor [TNF]-α). Our synthesis of results through meta-analysis may contribute toward developing biomarkers for risk prediction in high-risk populations.

## Materials and Methods

We conducted this meta-analysis according to the guidelines stated in the Meta-Analysis of Observational Studies in Epidemiology (MOOSE) statement (14). We provide a completed MOOSE checklist as supplementary material (Supplementary Table 1, available online).

### Literature Search Strategy

We executed a literature search in MEDLINE, EMBASE, and Web of Science to comprehensively capture publications with dates starting from inception (1966, 1946, and 1900, respectively) of the databases to January 1, 2017. We searched the databases to identify observational studies with prospectively collected data on serological immune markers and incident NHL. Our article search strategy used controlled database vocabulary where applicable, key words, and boolean logic to apply the following search terms and logic: “‘non-hodgkin lymphoma’ AND (‘interleukin 6’ OR ‘interleukin 10’ OR ‘tumor necrosis factor alpha’ OR ‘cxcl13’ OR ‘cd23 antigen’ OR ‘cd27 antigen’ OR ‘cd30 antigen’).” No other restrictions were imposed on the search. We sought additional articles from the reference lists of articles identified through the database search and of recent review articles (3,12,13), as well as from unpublished studies presented at national meetings with permission from willing investigators. A library information science specialist was consulted regarding database coverage and implementing controlled search vocabulary.

### Inclusion and Exclusion Criteria

Studies were included in this meta-analysis if they met the following criteria: (1) studies with prospective collection of plasma or serum for measurement of immunological biomarkers; (2) original articles reporting odds ratios (OR), hazard ratios, rate ratios, or relative-risks as measures of association, or data from which an estimate of the OR could be approximated; (3) studies that reported the association between any subset of prediagnosis serum biomarkers of interest and NHL risk or the risk of subtypes of NHL as outcomes; and (4) studies that reported estimates adjusted or controlled for a minimum of age and sex, but not other biomarkers. For studies of HIV-infected participants, adjustment criteria included receipt of highly active antiretroviral therapy (HAART) and at least one marker of immunological function (e.g. CD4+ cell counts or duration of infection). We excluded case reports, conference abstracts, and review articles.

### Data Items and Data Extraction Strategy

The following data were extracted from each publication: the biomarker(s) being assessed, NHL outcome including subtypes, timing of blood draw prior to NHL diagnosis (prediagnosis time lag), HIV serostatus, HAART exposure, adjustment variables, sample size (counts of cases and controls), country where the study was conducted, the first author’s name, publication year, and estimates of measures of association with their corresponding 95% confidence intervals (CIs) or standard errors for each comparison evaluated, and the document identification number for the publication. We also extracted the boundaries of predictor categories when biomarkers were analyzed as categorical predictors. Two of the co-authors (RSB and SBM) extracted results and information from the manuscripts of eligible studies onto spreadsheets, but without double entry. These authors (RSB and SBM) verified the accuracy of the collected data through cross-inspection of entered data. Discordant findings were resolved by discussion and consensus between the authors.

### Data Analysis

#### 

***Data Harmonization of Published Results.*** Since all studies reported ORs, we natural log-transformed the ORs and estimated the standard errors of the log-ORs by taking the natural logarithm of the upper and lower bound of the 95% confidence intervals, then dividing the difference by 3.92 (twice the 97.5th percentile of the standard normal distribution) (15). Many publications (16–22) had analyzed their predictor biomarkers on a continuous natural logarithm unit scale, or on a continuous scale that could be rescaled to be commensurate with natural logarithm units. For publications (8–11,13,23–27) presenting ORs estimated with categorized predictor biomarkers, we first applied a log-transformation to the category boundaries and calculated the intracategory midpoints. Using a published SAS macro (28), we applied a multistep procedure (29,30) that included fitting an inverse-variance weighted regression on the log-OR over the midpoints of biomarker categories. This allowed us to obtain an estimate of the change in log-odds of NHL for each logarithm-unit change in each biomarker, and its corresponding standard error, had the predictor not been categorized in the published analysis. For publications (9,26) that did not present the category boundaries for biomarkers categorized by percentiles, we first estimated the predictor biomarker percentiles assuming a normally distributed natural log-transformed biomarker with the mean and the standard deviation estimated from available statistics of the distribution using methods previously described (31,32).

Considering studies that estimated associations within strata defined by prediagnosis time lag, we collapsed the strata by calculating the inverse-variance weighted average of log-odds ratios over the time intervals to produce estimates of biomarker-NHL associations for the composite overall NHL outcome averaged over the maximum range of prediagnosis lag time, as well as within broader categories of early prediagnosis time lag (defined as 6 to 10 or more years prior to diagnosis), and late prediagnosis time lag (0–5 years prior to diagnosis, 0 being within the year of diagnosis). We also averaged results for NHL subtype outcomes by groups of subtypes, including diffuse large B-cell lymphoma (DLBCL), chronic lymphocytic leukemia/small lymphocytic lymphoma/prolymphocytic leukemia (CLL/SLL/PLL), and follicular lymphoma (FL), all aggregated according to Surveillance, Epidemiology, and End Results Program (SEER) International Classification of Diseases for Oncology third edition (ICD-O-3) morphology codes (33).

#### 

***Estimation of Meta-Analytic Summary ORs.*** Anticipating between-study heterogeneity a priori, we fit a restricted maximum likelihood random-effects model (34) to calculate summary ORs across studies for each biomarker. We also stratified the analyses by subgroups of HIV-serostatus and contrasted the OR estimates across serostatus subgroups by estimating a ratio-of-odds-ratios (RORs) and corresponding 95% confidence intervals and *P* values. Similarly, we calculated pairwise RORs and their corresponding 95% confidence intervals with *P* values from z-tests to compare the OR estimates between pairs of histological subtypes of NHL. In addition, to the extent possible, we carried out stratified analyses within strata defined by HAART exposure and prediagnosis time lag ranges (0–5 years and 6–10 years prior to NHL diagnosis).

#### 

***Estimation of Between-Study Heterogeneity.*** We assessed the presence of statistical heterogeneity between studies by conducting Cochran's *Q* test for statistical heterogeneity. Cochran's *Q* test statistic is computed as the sum, over all studies, of the squared deviation of each log-OR from the overall summary estimate weighted by the variance for the given log-OR (35). The *Q* test statistic follows a χ^2^ distribution with *k*-1 degrees of freedom (where *k* is the number of studies). We chose a statistical significance threshold of a two-sided *P* value less than .1 to indicate the presence of heterogeneity (35). We also calculated Higgins' *I^2^*, a measure of statistical heterogeneity, as the proportion of between-study variance relative to overall variance (overall variance being the sum of between-study and within-study variance) across the observed study log-ORs (36). *I*^2^ ranges from 0% for no heterogeneity to 100%, with *I*^2^ less than 25% indicating low heterogeneity, *I*^2^ between 25% and 75% inclusive indicating moderate heterogeneity, and *I*^2^ greater than 75% signifying high heterogeneity (37).

#### 

***Assessment of Publication Bias and Influential Data.*** We assessed publication bias by visual inspection of funnel plots (38) of the meta-analytic summary estimates of ORs plotted against their respective standard errors for each biomarker included in our study. An asymmetric distribution of the plotted points exceeding the 90% pseudo-confidence interval of the funnel plot indicate potential presence of publication bias. We also ran Egger's regression tests for each funnel plot with *P* value less than .1, signaling the presence of potential publication bias (39). Furthermore, we quantified the potential effect of publication bias on our results using trim-and-fill analyses described by Duval and Tweedie (40,41). Trim-and-fill analyses first estimate the results of hypothetically unreported studies using the observed set of study results, such that the asymmetric part of the funnel plot is filled. Then, outlying study estimates are excluded (“trimmed”) from outside of the funnel plot pseudo-confidence intervals. Finally, meta-analytic summaries are re-estimated including the estimated hypothetically unpublished results to see if they substantially alter final summary estimates.

Lastly, we do not include formal assessments of publication quality in our analyses because, after applying our inclusion criteria, we expect limited variation in the quality of prospective studies retrieved and such assessments of quality have been shown to have limited utility in mitigating bias in estimation of associations (42).

We constructed the final analytic datasets in SAS version 9.4 (Cary, NC). Statistical analyses were implemented in R version 3.2.2 (43) with the *meta* and *metafor* packages (44,45).

## Results

### Study Selection

**Figure 1.**
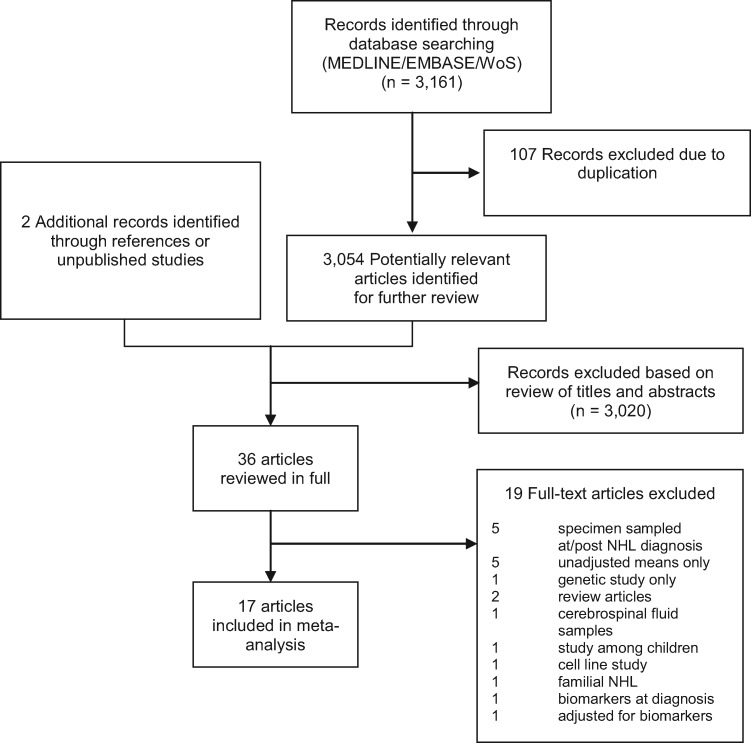
Flowchart for systematic literature search and selection of studies of circulating biomarkers and NHL risk. NHL = non-Hodgkin lymphoma.

The flow diagram of our literature search is shown in Figure 1, with details of the included set of 17 English language papers (no foreign language papers were captured by our search) provided in Table 1. We further excluded one study (21) from the analyses of IL-6 and IL-10, but retained it for other analyses, because the cases and controls completely overlapped with those of another study (17). Other included studies nested within the same parent cohorts had at most only partial, but not complete, overlap of study subjects and, therefore, were included here without modification. For IL-10 analyses, we further excluded another study (17) because it categorized biomarker levels as detectable versus undetectable. Our included studies comprised a total of 8684 participants (4047 cases, ignoring sample overlap, of which 11% were HIV-infected, and 4637 control subjects, of which 13% were HIV-infected), and considered biomarkers sampled over a long range of time intervals from within the year of diagnosis to up to 23 years prior to NHL diagnoses (Table 1).

**Table 1.** Characteristics of 17 prospective studies included in the meta-analysis

Source	Year*	Location, cohort, enrollment years†	Sex	Age, y‡	Biomarker(s)	Relevant NHL subtypes	Cases	Control subjects	HIV sero-status	Pre-NHL time interval§	Covariates‖
Purdue et al. (23)	2009	United States, PLCO, 1993–2001	M/F	55–74	sCD30	B-NHL, CLL/SLL, DLBCL, FL	234	234	HIV-	1–10	Matched: age (baseline), sex, race, blood draw date (baseline), center
Gu et al. (24)	2010	United States, NYUWHS, 1985–1991	F	35–65	IL-10, IL-6, TNF-α	B-NHL	92	184	HIV-	0–≥15	Matched: age, race, blood draw date; BMI, alcohol intake, smoking
Saberi Hosnijeh et al. (11)	2010	Italy, EPIC Italy, 1993–1998	M/F	35–65	IL-10, IL-6, TNF-α	B-NHL	86	86	HIV-	0–10	Matched: age (diagnosis), age (baseline), recruitment (baseline) date, sex, center; BMI, alcohol intake
Breen et al. (17)	2011	United States, MACS, 1984–1985/1987–1991	M	24–60	IL-6, sCD23, sCD27, sCD30	B-NHL, DLBCL	179	179	HIV+	0–5	Matched: duration of HIV infection/duration since study entry date, expected sample availability; age, CD4+ T-cells/mm^3^
Purdue et al. (25)	2011	United States, PLCO, 1993–2001	M/F	55–74	IL-10, IL-6, TNF-α, sCD27	B-NHL, CLL/SLL, DLBCL, FL	297	297	HIV-	1–10	Matched: age (baseline), sex, race, blood draw date (baseline), center
Rabkin et al. (18)	2011	United States, NCI, 1985–2004	M/F	29–44	IL-10, IL-6, TNF-α	B-NHL	63	181	HIV+	0.1–2	Matched: age (diagnosis), race, sex, blood draw date (period), CD4+ T-cells/mm^3^ (diagnosis), cohort, sample type
Vermeulen et al. (26)	2011	Italy, EPIC Italy, 1993–1998	M/F	35–70	sCD30	B-NHL	35	36	HIV-	2–≥6	age (baseline), sex; BMI
De Roos et al. (19)	2012	United States, WHI OS, 1994–1998	F	50–79	CXCL13, sCD23, sCD27, sCD30	B-NHL, CLL/SLL/PLL, DLBCL, FL	491	491	HIV-	0–13	Matched: age (birth year), blood draw date (baseline), region
Conroy et al. (10)	2013	United States, MEC Biospecimen Subcohort, 2001–2006	M/F	45–75	IL-10, IL-6, TNF-α	B-NHL, DLBCL, FL	272	541	HIV-	0–11.5	Matched: age, sex, race, region (state), blood draw date and time, fasting hours (pre-blood draw)
Hussain et al. (20)	2013	United States, WIHS, 1994–1995/2001–2002	F	<30– ≥50	CXCL13, IL-6, sCD23, sCD27, sCD30	B-NHL	22	78	HIV+	0.1–4.7	Matched: age, race, CD4+ T-cells/mm^3^, duration since seroconversion; HIV viral load, HAART, smoking, HCV, education
Purdue et al. (9)	2013	United States, PLCO, 1993–2001	M/F	55–74	CXCL13, IL-10, IL-6, TNF-α	B-NHL, CLL/SLL, DLBCL, FL	301	301	HIV-	5–13	Matched: age (baseline), sex, race, center, blood draw date and time
Edlefsen et al. (22)	2014	United States, WHI OS, 1994–1998	F	50–79	IL-10, IL-6, TNF-α	B-NHL, CLL/SLL/PLL, DLBCL, FL	491	491	HIV-	<3–13	Matched: age, blood draw date, region
Vendrame et al. (21)	2014	United States, MACS, 1984–1985/1987–1991	M	24–70	IL-10, IL-6, TNF-α	B-NHL	176	176	HIV+	0–5	Matched: duration of HIV infection/duration since study entry date, expected sample availability; age, CD4+ T-cells/mm^3^
Bassig et al. (8)	2015	Shanghai, SWHS, 1996–2000; Shanghai, SCS, 1986–1989; Singapore, SCHS, 1993–1998	M/F	40–74	sCD27, sCD30	B-NHL	218	218	HIV-	0–≥10	SCS: age (baseline), sex, blood draw date, region (neighborhood); SCHS: age (baseline), sex, baseline date, biospecimen collection date, dialect; SWHS: age (baseline), blood draw date; age, smoking
Purdue et al. (27)	2015	Finland, ATBC, 1985–1988	M	50–69	sCD23, sCD27, sCD30	B-NHL, CLL/SLL, DLBCL	272	325	HIV-	2–23	Matched: age (baseline), blood draw date, number of prior specimen thaws; smoking
Hosnijeh et al. (13)	2016	Italy, EPIC Italy, 1993–1998; Sweden, NSHDS/VIP, 1985–2008	M/F	35–70	sCD27, sCD30	B-NHL, CLL/SLL, DLBCL, FL	218	218	HIV-	1–17	Matched: age (baseline), sex, blood draw date, cohort, center
Epstein et al. (16)	2018	United States, NHS 1989–1990; HPFS, 1993–1994	M/F	30–75	CXCL13, IL-10, IL-6, TNF-α, sCD30	B-NHL, CLL/SLL, DLBCL, FL	600	601	HIV-	0–≥10	Matched: age (blood draw), race, blood draw time of day, cohort

* Year original article was published. ATBC = Alpha-Tocopherol, Beta Carotene Cancer Prevention; B-NHL = B-cell non-Hodgkin lymphoma; BMI = body mass index (in kg/m^2^); CLL/SLL/PLL = chronic lymphocytic leukemia/small lymphocytic lymphoma/prolymphocytic leukemia; DLBCL, diffuse large B-cell lymphoma; EPIC Italy = Italian European Prospective Investigation into Cancer and Nutrition Cohort; FL = follicular lymphoma; HAART = highly active antiretroviral therapy; HPFS = Health Professionals Follow-up Study; MACS = Multicenter AIDS Cohort Study; MEC = Multiethnic Cohort; NCI = US National Cancer Institute; NHL = non-Hodgkin lymphoma; NHS = Nurses’ Health Study; NSAID = Nonsteroidal anti-inflammatory drug; NSHDS = Northern Sweden Health and Disease Study; NYUWHS = New York University Women's Health Study; PLCO = Prostate, Lung, Colorectal, and Ovarian Cancer Screening Trial; SCHS = Singapore Chinese Health Study; NYUWHS = New York University Women's Health Study; PLCO = Prostate, Lung, Colorectal, and Ovarian Cancer Screening Trial; SCS = Shanghai Cohort Study; SWHS = Shanghai Women’s Health Study; VIP = Västerbotten Intervention Program; WHI OS = Women’s Health Initiative Observational Study component; WIHS = Women’s Interagency HIV Study.

† Country or city, nesting cohort study name, and enrollment period of nesting cohort study. Years reported for Rabkin (18) were years of NHL diagnosis in combined NCI cohort data.

‡ Age at enrollment into the nesting cohort study. Where enrollment age not reported, age range from article descriptive statistics provided.

§ Pre-NHL time interval refers to the range of time intervals, in years, prior to NHL diagnosis wherein venipuncture and blood sample collection was conducted. Lower bound of 0 means within the year of, but prior to, NHL diagnosis.

‖ Matching factors listed defining matching sets used in conditional regression; additional covariates included in models listed after semicolon. Otherwise, covariates for unconditional logistic regression listed for some studies (16, 26). Covariates listed are for the analyses of the composite NHL outcome. Analyses for subtype outcomes may have used different models (eg, polytomous logistic regression) and adjusted for additional sets of covariates.

¶ Subjects for Rabkin (18) comprised a combination of three HIV-infected cohorts followed at the US National Cancer Institute (NCI).

### Meta-Analyses

**Figure 2.**
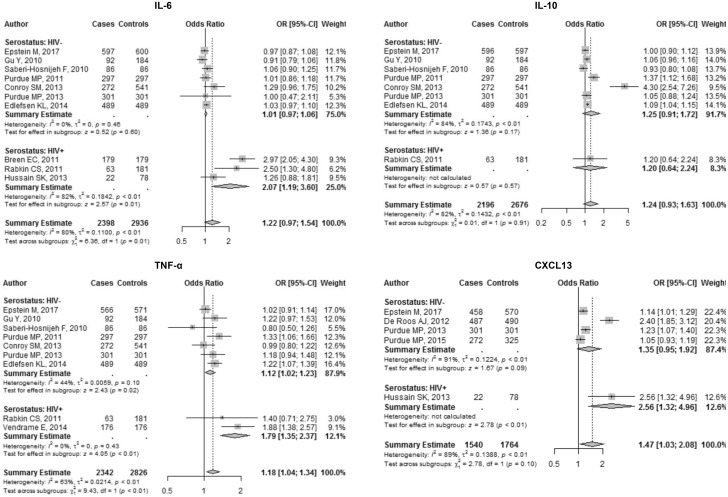
Forest plots for cytokines and chemokine. Odds ratio (OR) point estimates represented by gray squares with error bars indicating 95% confidence intervals (CIs); the size of the squares is proportional to the precision weight of each study in the random-effects meta-analysis. Diamonds indicate the summary ORs calculated from a random-effects model, with the width denoting the 95% CIs.

***Interleukin-6*** ***.*** Ten studies assessed associations between IL-6 levels and NHL. Each natural log-unit increase in circulating IL-6 was associated, though not statistically significantly, with a 22% increase in odds of NHL (OR = 1.22, 95% CI = 0.97 to 1.54) (Table 2, Figure 2). In serostatus subgroup analyses, the summary OR estimate was higher among HIV-infected subjects (OR = 2.07, 95% CI = 1.19 to 3.60) compared to HIV-uninfected subjects (OR = 1.01, 95% CI = 0.97, 1.06), with evidence of a difference between the two estimates (P < .001) (Table 2, Figure 2). When considering NHL subtypes (Table 3, Supplementary Figure 1, available online), we find that levels of circulating IL-6 had a modest association with DLBCL, and pairwise comparisons of follicular lymphoma versus DLBCL showed a modest difference (Table 3).

**Table 2.** Meta-analysis results for B cell NHL overall and by HIV serostatus

	All subgroups					HIV serostatus subgroups					
				*Q* Test†		HIV+		HIV-		Meta-regression ROR‡ (95% CI) for HIV+ / HIV-	*P*‡
Biomarker	No.	OR (95% CI)	I^2^* (95% CI)	*Q*-statistic	*P*	OR (95% CI)	*I*^2^ (95% CI)	OR (95% CI)	*I*^2^ (95% CI)		
IL-6	10	1.22 (0.97 to 1.54)	80 (65 to 89)	45.61	<.001	2.07 (1.19 to 3.60)	82 (44 to 94)	1.01 (0.97 to 1.06)	0 (0 to 69)	1.96 (1.53 to 2.50)	<.001
IL-10	8	1.24 (0.93 to 1.63)	82 (65 to 90)	38.43	<.001	1.20 (0.64 to 2.24)	—^§^	1.25 (0.91 to 1.72)	84 (69 to 92)	0.96 (0.33 to 2.83)	.943
TNF-α	9	1.18 (1.04 to 1.34)	63 (23 to 82)	21.46	.035	1.79 (1.35 to 2.37)	0 (– to –)	1.12 (1.02 to 1.23)	44 (0 to 76)	1.58 (1.15 to 2.18)	.005
CXCL13	5	1.47 (1.03 to 2.08)	89 (78 to 95)	37.00	<.001	2.56 (1.32 to 4.96)	—	1.35 (0.95 to 1.92)	91 (79 to 96)	1.89 (0.69 to 5.23)	.218
sCD23	4	1.57 (1.21 to 2.05)	90 (77 to 96)	29.24	<.001	1.59 (1.23 to 2.06)	0 (– to –)	1.58 (0.93 to 2.66)	0 (0 to 69)	1.00 (0.54 to 1.87)	.996
sCD27	7	2.18 (1.20 to 3.98)	92 (87 to 96)	79.67	<.001	4.93 (3.00 to 8.08)	0 (– to –)	1.61 (0.89 to 2.93)	84 (69 to 92)	3.35 (1.05 to 10.71)	.041
sCD30	9	1.65 (1.22 to 2.22)	90 (84 to 94)	83.01	<.001	3.69 (2.40 to 5.69)	11 (– to –)	1.40 (1.11 to 1.76)	44 (0 to 76)	2.55 (1.38 to 4.73)	.003

* Higgins’ I^2^ statistic measuring the proportion of the observed variance between studies relative to the total variance of a set of studies. CI = confidence interval; OR = odds ratio; ROR= ratio of odds ratios.

† Q test assessing the degree to which study effect sizes are concordant.

‡ The ratio of odd-ratios compares the odds-ratio for the HIV+ subgroup with that of the HIV- subgroup (HIV+/HIV-). The corresponding *P* values are computed from a test of the null hypothesis of no difference between the serostatus groups.

§ “—” and “–” denote Higgins’ I^2^ statistics and confidence intervals that were not calculated because of inadequate sample size, n = 1 and n = 2, respectively.

**Table 3.** Meta-analysis results for B cell NHL subtypes

							Comparison of summary ORs‡			
				*Q* test†		Summary OR	DLBCL		Follicular lymphoma	
Biomarker	Outcome	No.	*I*^2^* (95% CI)	Statistic	*P*	OR (95% CI)	ROR (95% CI)	*P*	ROR (95% CI)	*P*
IL-6	CLL/SLL/PLL	4	0 (0 to 0)	0.19	.996	0.98 (0.92 to 1.06)	1.15 (0.99 to 1.34)	.074	0.97 (0.87 to 1.09)	.652
	DLBCL	6	0 (0 to 74)	4.80	.570	1.13 (0.99 to 1.30)	1.00 (reference)		0.85 (0.72 to 1.00)	.044
	Follicular lymphoma	5	9 (0 to 81)	4.41	.492	0.96 (0.88 to 1.05)	–		1.00 (reference)	
IL-10	CLL/SLL/PLL	4	78 (41 to 92)	13.76	.008	1.09 (0.88 to 1.34)	1.04 (0.83 to 1.29)	.747	1.01 (0.81 to 1.26)	.955
	DLBCL	5	45 (0 to 80)	7.28	.201	1.13 (1.06 to 1.21)	1.00 (reference)		0.97 (0.87 to 1.07)	.485
	Follicular lymphoma	5	66 (13 to 87)	11.93	.036	1.09 (1.02 to 1.18)	–		1.00 (reference)	
TNF-α	CLL/SLL/PLL	4	0 (0 to 66)	1.34	.854	1.15 (1.04 to 1.27)	0.91 (0.73 to 1.14)	.410	1.21 (0.89 to 1.65)	.214
	DLBCL	5	62 (0 to 86)	10.41	.064	1.04 (0.85 to 1.28)	1.00 (reference)		1.34 (0.94 to 1.90)	.107
	Follicular lymphoma	5	66 (12 to 87)	11.82	.037	1.39 (1.04 to 1.86)	–		1.00 (reference)	
CXCL13	CLL/SLL/PLL	4	77 (36 to 91)	12.81	.012	1.43 (0.97 to 2.11)	1.18 (0.65 to 2.12)	.584	1.20 (0.61 to 2.37)	.604
	DLBCL	4	85 (61 to 94)	19.43	.001	1.69 (1.08 to 2.62)	1.00 (reference)		1.02 (0.50 to 2.08)	.964
	Follicular lymphoma	3	86 (60 to 95)	14.38	.002	1.71 (0.98 to 3.00)	–		1.00 (reference)	
sCD23	CLL/SLL/PLL	2	99 (97 to 99)	69.59	.000	2.62 (0.74 to 9.19)	0.48 (0.14 to 1.69)	.253	0.75 (0.21 to 2.71)	.664
	DLBCL	3	49 (0 to 85)	3.90	.272	1.25 (1.11 to 1.41)	1.00 (reference)		1.57 (1.19 to 2.08)	.001
	Follicular lymphoma	1	—^§^	0.00	1.000	1.97 (1.53 to 2.53)	–		1.00 (reference)	
sCD27	CLL/SLL/PLL	3	95 (89 to 98)	39.81	<.001	2.03 (0.73 to 5.64)	1.06 (0.29 to 3.83)	.927	1.08 (0.22 to 5.16)	.927
	DLBCL	4	89 (74 to 95)	26.90	<.001	2.15 (0.99 to 4.67)	1.00 (reference)		1.01 (0.25 to 4.18)	.985
	Follicular lymphoma	2	94 (81 to 98)	16.56	<.001	2.18 (0.67 to 7.16)	–		1.00 (reference)	
sCD30	CLL/SLL/PLL	4	76 (35 to 91)	12.70	.013	1.23 (1.05 to 1.44)	1.38 (0.84 to 2.26)	.205	1.89 (1.07 to 3.35)	.028
	DLBCL	5	88 (74 to 94)	32.94	<.001	1.69 (1.06 to 2.71)	1.00 (reference)		1.37 (0.67 to 2.82)	.387
	Follicular lymphoma	3	87 (64 to 96)	15.66	.001	2.33 (1.35 to 4.01)	–		1.00 (reference)	

* Higgins' *I*^2^ statistic measuring the proportion of the observed variance between studies relative to the total variance of a set of studies. CI = confidence interval; CLL/SLL/PLL = chronic lymphocytic leukemia/small lymphocytic lymphoma/prolymphocytic leukemia; DLBCL = diffuse large B-cell lymphoma.

† *Q* test assessing the degree to which study effect sizes are concordant.

‡ ORs and *P* values for comparisons of estimates between outcomes for each biomarker. Each ROR compares the odds ratio for the column biomarker to that of the row biomarker as reference, for example for IL-6 OR_DLBCL_/OR_CLL/SLL/PLL_=1.15, with corresponding Wald-type confidence interval computed using the square root of the sum of the OR variances.

§ Em dash “—” denotes statistics that were not calculated because of inadequate sample size. En dash “–” indicates omitted results comparing DLBCL to Follicular lymphoma which are exact inverses of results comparing Follicular lymphoma to DLBCL in the subsequent colum.

***Interleukin-10*** ***.*** A total of eight nested case-control studies assessed associations between circulating IL-10 levels and NHL. Our summary estimate (OR = 1.24, 95% CI = 0.93 to 1.63) suggests that each natural log-unit increase in circulating IL-10 is associated with a nonstatistically significant increase of 24% in the odds of NHL (Table 2, Figure 2). Among HIV-infected subjects, we found a moderate association with a wide confidence interval (OR = 1.20, 95% CI = 0.64 to 2.24), as well as among HIV-uninfected subjects (OR = 1.25, 95% CI = 0.91 to 1.72), with no meaningful difference between the two estimates (*P* = .943) (Table 2, Figure 2). DLBCL and follicular lymphoma showed associations with elevated IL-10 levels, and we observed no substantial differences in estimates when conducting pairwise comparisons by subtype (Table 3, Supplementary Figure 1, available online).

***Tumor Necrosis Factor-α***. A set of nine studies assessed associations between TNF-α levels and NHL. The overall summary estimate of OR = 1.18 (95% CI = 1.04 to 1.34) (Table 2, Figure 2) illustrates that elevated serum levels of TNF-α are associated with increased risk of NHL overall, increasing the odds by 18% per natural log unit. When comparing estimates between HIV-infected (OR = 1.79, 95% CI = 1.35 to 2.37) and HIV-uninfected (OR = 1.12, 95% CI = 1.02 to 1.23), we found evidence of a difference in ORs between HIV serostatus groups (*P* = .005) (Table 2, Figure 2). Analyses within NHL subtypes showed evidence of associations between TNF-α and CLL/SLL/PLL only, with no differences found in pairwise comparisons between subtypes (Table 3, Supplementary Figure 1, available online).

***CXCL13*** ***.*** Five studies in total assessed associations between CXCL13 levels and NHL. A summary estimate of OR = 1.47 (95% CI = 1.03 to 2.08) (Table 2, Figure 2) shows that each natural log-unit increase in circulating CXCL13 is associated with a 47% increase in odds of NHL. When assessed by serostatus subgroups, the summary OR estimate among HIV-infected subjects was OR = 2.56 (95% CI = 1.32 to 4.96) compared to OR = 1.35 (95% CI = 0.95 to 1.92) among HIV-uninfected subjects with no evidence of a difference by serostatus (Table 2, Figure 2). DLBCL was the only subtype to show an association with NHL with some statistical confidence, and pairwise comparisons by subtype showed no meaningful differences (Table 3, Supplementary Figure 1, available online).

**Figure 3.**
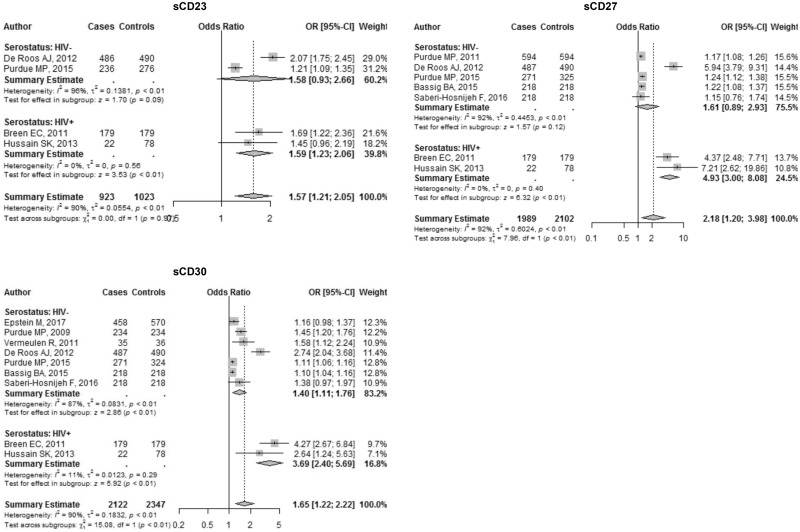
Forest plots for soluble receptors. Odds ratio (OR) point estimates represented by gray squares with error bars indicating 95% confidence intervals (CIs); the size of the squares is proportional to the precision weight of each study in the random-effects meta-analysis. Diamonds indicate the summary ORs calculated from a random-effects model, with the width denoting the 95% CIs. WoS = Web of Science.

***Soluble CD23, CD27,*** ***and*** ***CD30*** ***.*** Soluble CD23, CD27, and CD30 had four, seven, and nine studies assessing its relationship with NHL, respectively. Overall, the meta-analytic estimates showed increased risk of NHL associated with sCD23 (OR = 1.57, 95% CI = 1.21 to 2.05), sCD27 (OR = 2.18, 95% CI = 1.20 to 3.98), and sCD30 (OR = 1.65, 95% CI = 1.22 to 2.22) (Table 2, Figure 3). When we compared HIV-infected versus uninfected subgroups, we observed differences in biomarker associations between NHL and both sCD27 and sCD30 (Table 2, Figure 3). Elevated levels of sCD23 were associated with DLBCL and follicular lymphoma, whereas all subtypes showed an association with elevated levels of sCD30 (Table 3, Supplementary Figure 1, available online). Pairwise comparisons of sCD23 associations with follicular lymphoma versus DLBCL showed evidence of differences; similarly, for sCD30, the comparison of its association with follicular lymphoma versus its association with CLL/SLL/PLL showed evidence of a meaningful difference. No other pairwise subtype differences were notable (Table 3).

***Prediagnosis Time Lag*** ***and*** ***HAART Exposure*** ***.*** We conducted analyses stratified by early (6–10 years prior to NHL diagnosis) versus late collection of biomarkers (0–5 years prior to NHL diagnosis) (Table 4). In the early period, elevated levels of IL-10 (OR = 1.10, 95% CI = 1.03 to 1.17), TNF-α (OR = 1.19, 95% CI = 1.05 to 1.34), and sCD30 (OR = 1.34, 95% CI = 1.00 to 1.80) were associated with NHL, whereas ORs and confidence intervals for other biomarkers indicated some positive but uncertain associations with NHL. In contrast, we observed comparatively higher OR in the late period for IL-6, TNF-α, CXCL13, sCD23, sCD27, and sCD30. Formal comparisons of ORs between the two prediagnosis time strata yielded no important differences. We were able to carry out analyses stratified by HAART exposure only for IL-6, sCD23, sCD27, and sCD30, with only one study (20) providing an estimate for HAART-exposed individuals (Supplementary Table 3, available online). Summary estimates were generally higher among HAART-unexposed individuals (estimates ranging from OR = 1.75, 95% CI = 1.30 to 2.36 to OR = 4.72, 95% CI = 2.81 to 7.93), whereas the OR estimates for the HAART-exposed group were generally lower, except for sCD27 for which the sample size was limited (n = 9 HAART exposed cases, n = 37 control subjects) resulting in potential sparse data bias. We also did not observe any evidence of meaningful differences in the OR estimates across HAART exposure strata.

**Table 4.** Results for all B-cell non-Hodgkin lyphoma (NHL) by prediagnosis time interval: comparing early versus late biomarker sample collection

						Pre-diagnosis time interval				
									Meta-regression	
		Early (6–≥10 y)*				Late (0–5 y)*			comparison of	
Analyte	No.	OR (95% CI)	*P*	*I*^2^ (95% CI)	N	OR (95% CI)	*P*	*I*^2^ (95% CI)	early vs late ROR (95% CI)	*P*
IL-6†	2	1.04 (0.89 to 1.21)	.645	0 (– to –)	6	1.44 (1.00 to 2.08)	.052	87 (74 to 93)	0.74 (0.39 to 1.40)	.352
IL-10	3	1.10 (1.03 to 1.17)	.003	0 (0 to 34)	6	1.12 (0.98 to 1.28)	.087	44 (0 to 78)	0.98 (0.84 to 1.15)	.817
TNF-α	2	1.19 (1.05 to 1.34)	.005	0 (– to –)	6	1.25 (1.01 to 1.54)	.038	85 (70 to 93)	0.96 (0.67 to 1.36)	.813
CXCL13	2	1.33 (0.67 to 2.62)	.411	96 (88 to 99)	2	2.69 (2.20 to 3.28)	<.001	0 (– to –)	0.50 (0.24 to 1.06)	.070
sCD23	2	1.41 (0.98 to 2.01)	.061	92 (74 to 98)	4	1.62 (1.21 to 2.15)	.001	89 (75 to 95)	0.87 (0.54 to 1.39)	.559
sCD27	4	1.50 (0.96 to 2.35)	.077	88 (71 to 95)	6	2.64 (1.34 to 5.21)	.005	96 (93 to 97)	0.58 (0.24 to 1.45)	.247
sCD30	4	1.34 (1.00 to 1.80)	.047	87 (69 to 95)	7	1.89 (1.28 to 2.79)	.001	94 (90 to 96)	0.73 (0.42 to 1.27)	.262

* The early category included studies that had categories with upper bounds of the time intervals that were greater than 10 years (e.g. intervals such as >7, 5–13, 9–13, 8–15, 15–23 years prior to diagnosis), whereas the late (0–5 year) category included studies with intervals that exceeded the upper bound of 5 years (e.g. 2–6, <7 years prior to diagnosis). For these analyses, estimates of associations covering both intervals completely, or nearly completely, were excluded. CI to confidence interval; OR to odds ratio.

† The IL-6 analyses included Vendrame, 2014 (21) and Breen, 2011 (17) which contain completely overlapping study subjects, but different assay technologies. We include them here but not in the manuscript because the results are not substantially different with or without exclusion, and given the small sample size, the additional information dominates the small bias because of the lack of independence for our assessment of associations by prediagnosis time periods and their differences.

***Heterogeneity*** ***.*** We found substantial heterogeneity in overall and subgroup (HIV serostatus, NHL subtypes) analyses. For analyses of the overall composite NHL outcome, all Cochran's *Q* tests indicated the presence of heterogeneity (i.e. all two-sided *P* values <.1), while Higgins' *I*^2^ values indicated moderate to large magnitudes of heterogeneity ranging from *I*^2^ = 63% (95% CI = 23% to 82%) to *I*^2^ = 91% (95% CI = 85% to 95%) (Table 2). When we conducted subgroup analyses within HIV-serostatus strata, heterogeneity measures decreased only modestly where calculable, with most *Q* tests indicating the presence of heterogeneity (Figures 2 and 3), and *I*^2^ proportions ranging from *I*^2^ = 44% (95% CI = 0% to 76%) to *I*^2^ = 96% (95% CI = 90% to 99%) within the HIV-uninfected subgroup. Within the HIV-infected group, sample sizes were small (at most n* *=* *3) rendering heterogeneity statistics unreliable. When we assessed associations by NHL histological subtypes, we found statistically detectable heterogeneity in two-thirds of comparisons (Cochran's *Q* tests <0.1), but with ranges of *I*^2^ statistics that were reduced compared to those of the composite NHL outcome (Table 3). We interpret these statistics with caution since the numbers of studies included in the analyses, particularly by subgroups, were limited relative to recommended sample sizes for these measures (46).

***Publication Bias*** ***and*** ***Influential Data*** ***.*** We provide a set of funnel plots for each analysis for our composite overall NHL outcome (Supplementary Figure 2, available online). Because of small sample sizes, evidence of symmetry in the distribution of meta-analytic summary ORs is inconclusive. Egger's regression tests suggest the presence of potential publication bias for the OR estimates of NHL for IL-6, IL-10, CXCL13, sCD27, and sCD30 (*P* < .1), although small samples limit the validity of this test. Trim-and-fill analyses indicated that studies predicted to be excluded from our analyses because of potential publication bias would have attenuated our estimates for all biomarkers, while maintaining the same direction of association (Supplementary Table 2, available online). Influence diagnostics show a few potentially influential studies: one study each for in the analyses for IL-6 (17), IL-10 (10), and CXCL13 (19) (Supplementary Figure S3, available online).

## Discussion

Two patterns become discernible from our analyses: (1) Elevated expression of immune stimulatory molecules, including cytokines, chemokines, and soluble receptors, precedes an NHL diagnosis, and (2) the associated increase in risk is generally higher among HIV-infected relative to HIV-uninfected individuals. These two inferences largely corroborate what has previously been reported in prior independent reports. These results also suggest that HIV itself, because of the immune dysregulation resulting from HIV, or the subtypes that primarily emerge in the presence of HIV, are key factors in the association between immune stimulatory molecules and NHL. Further, our study findings support the use of these molecules as biomarkers for an immune environment that promotes NHL.

IL-6 is a pluripotent cytokine that can stimulate B-cell proliferation and differentiation, foster cell survival, and promote tumor growth (47,48). IL-6 has also been linked to pro-inflammatory and Th17 immune responses, which are related to autoimmunity (49,50) and closely related to risks for NHL (51). We found that the positive association between IL-6 and NHL was stronger among HIV-infected compared to HIV-uninfected subjects, suggesting a modifying effect of HIV infection. The stronger associations between IL-6 and NHL among HIV-infected subjects could also be influenced by the higher proportion of the DLBCL histological subtype in the presence of HIV (2,52–54), a subtype that displayed the highest OR in our histological subtype-specific analyses for IL-6, particularly when compared to follicular lymphoma. Although these findings present with a high level of heterogeneity, they are nonetheless qualitatively consistent with the hypothesized etiologic role of IL-6 in the development of NHL.

IL-10 is a pleiotropic cytokine with stimulatory effects on B-cells and is suspected of inducing lymphomagenesis by promoting chronic B-cell activation (55–57). In a mouse model, IL-10 was required for the progression of B-cell lymphoma (58), and in humans, malignant NHL cells produce IL-10 (59,60). A growing body of literature, as described in a recent meta-analysis, showed that IL-10 gene polymorphisms, especially 3575 T/A and 1082 A/G, were associated with increased NHL risk or its subtypes, including DLBCL and follicular lymphoma (61–64). Our analyses of NHL subtypes corroborate results from studies of genetic polymorphisms because our study also found an association between IL-10 and DLBCL, as well as follicular lymphoma, lending credence to the hypothetical function of IL-10 in lymphomagenesis.

TNF*-*α is a potent pro-inflammatory cytokine that can induce B-cell activation, growth, differentiation, apoptosis, and chemotaxis (65–67). Knockout mouse models of *TNF* (68), as well as genetic association studies in humans (56,69,70), provide evidence of the involvement of TNF*-*α in lymphomagenesis. A potential mechanism through which TNF-α is involved in lymphomagenesis is enhancement of B-cell survival, differentiation, and proliferation mediated by the nuclear transcription factor (NF)-κB pathway (56,66). We found a higher summary OR estimate for NHL among the HIV-infected subgroup compared to the HIV-uninfected group, indicating that elevated levels of TNF-α confer higher risk of NHL in the context of HIV infection. In addition, we found evidence of associations between elevated levels of TNF-α and DLBCL and follicular lymphoma subtypes. These results are consistent with a hypothesized etiologic function of elevated TNF-α levels prior to the onset of NHL.

CXCL13 and its receptor, CXCR5, are required for B-cell homing to follicles in lymph nodes (71), suggesting that aberrant CXCL13 expression may be involved in the pathogenesis of B-cell lymphoma through abnormal chemotaxis of B-cells to tissues or abnormal B-cell activation (72). In addition, overexpression of the receptor-ligand pair CXCR5/CXCL13 has been observed in B-cell chronic lymphocytic leukemia (73), and follicular lymphoma cells have been seen to secrete CXCL13 (74). We found an association between NHL and elevated levels of CXCL13, and although our data were insufficient to reliably compare the CXCL13 and NHL associations across serostatus groups, we observed a markedly stronger association among HIV-positive versus HIV-negative individuals. In addition, DLBCL, a subtype more prevalent among HIV-infected populations, showed an association with elevated CXCL13 in our study. These results indicate a possible role for CXCL13 in lymphomagenesis, particularly in the context of HIV infection.

CD23, a cell-surface receptor for the Fc portion of IgE, can be proteolytically cleaved from the B-cell surface to produce its soluble form (sCD23) (75). Through the stimulatory action of IL-4, IL-13, and infectious agents (76), activated B-cells upregulate their expression and cleavage of CD23, subsequently increasing concentrations of sCD23 in serum. Serum sCD23 affects further B-cell stimulation including increases in IL-4-mediated IgH class switch recombination (75,77), potentially leading to aberrant recombination, which is implicated in lymphomagenesis. Additionally, sCD23 may also upregulate monocyte production of IL-6 (78), thereby increasing the likelihood of the development of various NHL subtypes in the context of autoimmune conditions (51). Contrasting the OR estimate for NHL among the HIV-infected group versus the HIV-uninfected group, we find no substantial differences, suggesting sCD23 may be a biomarker of NHL regardless of the presence or absence of HIV. Elevated levels of sCD23 were associated with DLBCL and follicular lymphoma, with a higher OR estimate for follicular lymphoma relative to DLBCL (*P* = .001) (Table 3), potentially suggesting a greater etiologic role for follicular lymphoma versus DLBCL.

CD27 and CD30 are members of the TNF-receptor superfamily (79,80). CD27 is involved in the activation of both T cells and B-cells, stimulating proliferation of T-cell proliferation (81) and inducing production of immunoglobulins by B-cells (82). CD30 was first discovered, and is frequently expressed, on Hodgkin lymphoma Reed-Sternberg cells. It is also found expressed on NHL cells, particularly in anaplastic large-cell lymphoma, but is less frequently expressed in cells of other NHL subtypes (83). CD30 is also expressed by activated T cells, which secrete cytokines that induce B-cell activation, differentiation, and proliferation (84,85). Cell membrane-associated CD27 and CD30 are proteolytically cleaved to produce the soluble forms of these molecules (sCD27 and sCD30) found in serum. Serum concentrations of both sCD27 and sCD30 have been elevated among those with viral infections and autoimmune diseases (86,87). The potential role of sCD27 in B-cell immunoglobulin production, and that of sCD30 in B-cell activation, implicates these molecules in lymphomagenesis. Similarly, our study found elevated levels of both sCD27 and sCD30 to be associated with NHL overall. Broken down by HIV serostatus groups, we found larger magnitudes of ORs among HIV-positive individuals relative to those who were HIV-negative, and although the estimates were imprecise because of limited sample sizes, this result aligns with prior findings that heightened concentrations of these biomarkers precede NHL, particularly during HIV infection (88,89). In our analyses by subtype, we found that sCD27 was associated with DLBCL and follicular lymphoma, and sCD30 showed an association with all NHL subtypes. Evidence of differences in OR estimates for follicular lymphoma versus DLBCL, and follicular lymphoma versus CLL/SLL/PLL, for sCD23 and sCD30, respectively, suggest that higher concentrations of these biomarkers may play a greater role in the development of follicular lymphoma relative to the other markers.

Temporal variations in the association between serum biomarkers and NHL may be due to etiologic factors or prodromal effects acting at different time intervals (17,20,89,90). We included exploratory analyses stratified by the early versus late collection of biomarkers. In the early period, we observed evidence of associations with NHL among several biomarkers (IL-10, TNF-α, sCD30) and notably stronger associations of several biomarkers (IL-6, TNF-α, CXCL13, sCD23, sCD27, sCD30) measured nearer in time to NHL diagnosis, although there were no meaningful differences between the two time intervals (Table 4). These findings are consistent with the inference that these biomarkers are elevated several years prior to NHL and that further increases in concentrations of these biomarkers may occur in the tumor microenvironment as clinical detectability of malignancy approaches.

Among the studies based in HIV-infected populations, the vast majority of cases and matched control subjects were HAART-naïve. Recently, serum levels of several immune markers, including IL-6, were shown to be elevated in HAART naïve individuals compared to those who were HIV-negative but normalized following HAART therapy (91). With the advent of HAART, the etiologic effect of HIV on NHL risk appears to have been attenuated, but not eliminated (3). In supplementary analyses, we assessed biomarker-NHL associations stratified by HAART exposure, and observed increased odds of NHL associated with higher elevations of biomarkers among the HAART unexposed relative to exposed groups for most biomarkers included in these analyses: IL-6, sCD23, sCD30 (Supplementary Table 3, available online). We note that these analyses are exploratory in nature because of limited sample sizes within each stratum (N = 1 for all HAART exposed; maximum N = 3 for HAART unexposed).

Major strengths of our study include the comprehensive coverage of literature and biomarkers with quantitative syntheses of results and the inclusion of studies with prospective collection of immune markers. Prior reports either included a limited set of biomarkers (13) or were descriptive in nature, thereby lacking quantitative summaries of published estimates (3,12). An additional strength of our study is that we included only studies that utilized a prospective-specimen collection, retrospective-blinded-evaluation (PRoBE) design with highly comparable control groups, thereby increasing our confidence in the validity of the reports. Furthermore, the use of multiplex assays in many of the included studies allowed several biomarkers to be analyzed and reported simultaneously, without regard to statistical significance, minimizing the “file-drawer” problem of studies hidden from publication because of results that were not statistically significant.

A weakness in our analyses is the modest number of studies for some biomarkers, which produced several limitations. First, sparse study counts limited our ability to adequately explore modifying factors across studies including prediagnosis time interval of biomarker collection, age, sex, and HAART exposure as potential modifiers of biomarker-NHL associations. We provide some exploratory analyses of associations by early versus late collection of biomarkers prior to NHL and stratified analyses by HAART exposure, but we note the substantial limitations of these analyses. For example, in the lag-time stratified analyses, there were overlapping time intervals over which biomarkers were collected such that the definitions of *early* versus *late* collection were not strictly mutually exclusive. Secondly, estimates of heterogeneity statistics, *I^2^* and *Q*, have been documented to be biased in small samples (46), and outliers tend to have higher influence in small samples. In addition, we did not find convincing evidence of potential publication bias partly because of the limited sample sizes that render funnel plots and Egger's regression *P* values unreliable (92), but also because simultaneous analyses of biomarkers from multiplex assays reduce the chance of nonsignificant associations going unpublished.

Another limitation of our study is the intrinsic variability in the biomarker quantitation among the studies in our analyses. We included studies that use various assay technologies, with biomarkers quantitated in different laboratories following different protocols and standards. Breen et al. (93) found considerable variability between multiple laboratory sites using high-sensitivity multiplex cytokine assays in their quantitation of 13 cytokines, across both study sites and multiplex assay technology, despite standardization of samples and laboratory protocols. Noble et al. (94) found significant variation in the quantitation of a standard cytokine provided to 11 laboratories, with the mean concentrations ranging between 67% and 136% of the grand mean. An additional contributing factor to heterogeneity in results is that we were unable to differentiate between germinal cell versus nongerminal cell lymphomas. Because these subtypes differ in etiologic mechanisms and in their interactions with the immune system (95–97), we expect these issues to contribute to the observed heterogeneity between studies, even within our subtype analyses because we were unable to further stratify by germinal cell origin.

Lastly, we acknowledge that our study is susceptible to bias because of multiple statistical testing of summary estimates and that multiple comparison adjustments to *P* values and confidence intervals widen our estimated confidence interval widths (98,99) and attenuate the magnitudes of the *P* values. However, these adjustments do not invalidate the overall qualitative message that, in general, levels of circulating markers are elevated prior to NHL diagnosis (Supplementary Table 4, available online).

In conclusion, our summaries concur with the general trends in published estimates and provide a systematic description of the variation in estimates of associations between NHL and expression of immune stimulatory molecules. Future research may further strengthen the inferences possible from a review such as ours by including larger sets of publications as the literature grows, particularly among HIV-infected populations, and pooled individual level data studies could allow for more robust control of confounding. Our findings provide support for the hypothesis that chronic immune activation is a crucial mechanism in lymphomagenesis; hence, its biomarkers could, in the future, have utility in developing models for early detection.

## Funding

This research was supported by the National Institutes of Health grant T32 CA09142; the Center for HIV Identification, Prevention, and Treatment (CHIPTS) NIMH grant P30MH058107; and the UCLA Center for AIDS Research (CFAR) grant 5P30AI028697, Core H.

## Notes

Affiliations of authors: Department of Epidemiology, Fielding School of Public Health, University of California, Los Angeles (UCLA), CA (SBM, RSB, CYJ, OAA, OM-M, SKH); Department of Medicine, Samuel Oschin Comprehensive Cancer Institute, Cedars-Sinai Medical Center, Los Angeles, CA (CYJ, SKH); Departments of Obstetrics and Gynecology and Microbiology, Immunology and Molecular Genetics, David Geffen School of Medicine at UCLA, Los Angeles, CA (OM-M); Department of Biostatistics, Fielding School of Public Health, University of California, Los Angeles, CA (REW); Cousins Center for Psychoneuroimmunology, Jane and Terry Semel Institute for Neuroscience and Human Behavior, Department of Psychiatry and Biobehavioral Sciences, David Geffen School of Medicine, University of California, Los Angeles, CA (ECB).

The ideas and opinions expressed herein are those of the authors, are not in any manner endorsed by the National Institutes of Health, the UCLA or their contractors and subcontractors.

The authors do not have any conflicts of interest to disclose.

## Supplementary Material

Supplementary Data
