# Supplementary material for: Markers of Immune Activation and Inflammation, and Non-Hodgkin Lymphoma: A Meta-Analysis of Prospective Studies
Source: JNCI Cancer Spectr. 2019 Mar 5;2(4):pky082. doi: 10.1093/jncics/pky082 (PMC6400235; doi:10.1093/jncics/pky082)
Supplement: Supplementary Data [file pky082_supp.docx]

**Supplementary Material**

**Supplementary Table S1:** MOOSE Checklist for Meta-analyses of Observational Studies

| **Item No** | **Recommendation** | **Reported on Page No** |
| --- | --- | --- |
| Reporting of background should include | | |
| 1 | Problem definition | 3 |
| 2 | Hypothesis statement | 3 |
| 3 | Description of study outcome(s) | 3 |
| 4 | Type of exposure or intervention used | 3 |
| 5 | Type of study designs used | 4 |
| 6 | Study population | 4-5 |
| Reporting of search strategy should include | | |
| 7 | Qualifications of searchers (e.g., librarians and investigators) | 1,4 |
| 8 | Search strategy, including time period included in the synthesis and key words | 4 |
| 9 | Effort to include all available studies, including contact with authors | 4 |
| 10 | Databases and registries searched | 4 |
| 11 | Search software used, name and version, including special features used (e.g., explosion) | 4 |
| 12 | Use of hand searching (e.g., reference lists of obtained articles) | 4 |
| 13 | List of citations located and those excluded, including justification | 28, Fig 1 |
| 14 | Method of addressing articles published in languages other than English | -- |
| 15 | Method of handling abstracts and unpublished studies | 4 |
| 16 | Description of any contact with authors | 4 |
| Reporting of methods should include | | |
| 17 | Description of relevance or appropriateness of studies assembled for assessing the hypothesis to be tested | 3-4 |
| 18 | Rationale for the selection and coding of data (e.g., sound clinical principles or convenience) | 5-6 |
| 19 | Documentation of how data were classified and coded (e.g., multiple raters, blinding and interrater reliability) | 5-6 |
| 20 | Assessment of confounding (e.g., comparability of cases and controls in studies where appropriate) | -- |
| 21 | Assessment of study quality, including blinding of quality assessors, stratification or regression on possible predictors of study results | -- |
| 22 | Assessment of heterogeneity | 6 |
| 23 | Description of statistical methods (e.g., complete description of fixed or random effects models, justification of whether the chosen models account for predictors of study results, dose-response models, or cumulative meta-analysis) in sufficient detail to be replicated | 6-7 |
| 24 | Provision of appropriate tables and graphics | 28+ |
| Reporting of results should include | | |
| 25 | Graphic summarizing individual study estimates and overall estimate | Fig 2, 3, Supplement |
| 26 | Table giving descriptive information for each study included | 28 |
| 27 | Results of sensitivity testing (e.g., subgroup analysis) | 31, 32, Supplement |
| 28 | Indication of statistical uncertainty of findings | 31, 32, Supplement |

*From*: Stroup DF, Berlin JA, Morton SC, et al, for the Meta-analysis Of Observational Studies in Epidemiology (MOOSE) Group. Meta-analysis of Observational Studies in Epidemiology. A Proposal for Reporting. *JAMA*. 2000;283(15):2008-2012. doi: 10.1001/jama.283.15.2008.

Transcribed from the original paper within the NEUROSURGERY® Editorial Office, Atlanta, GA, United Sates. August 2012.

**Supplementary Figure S1:** Forest Plots for NHL Subtype Outcomes


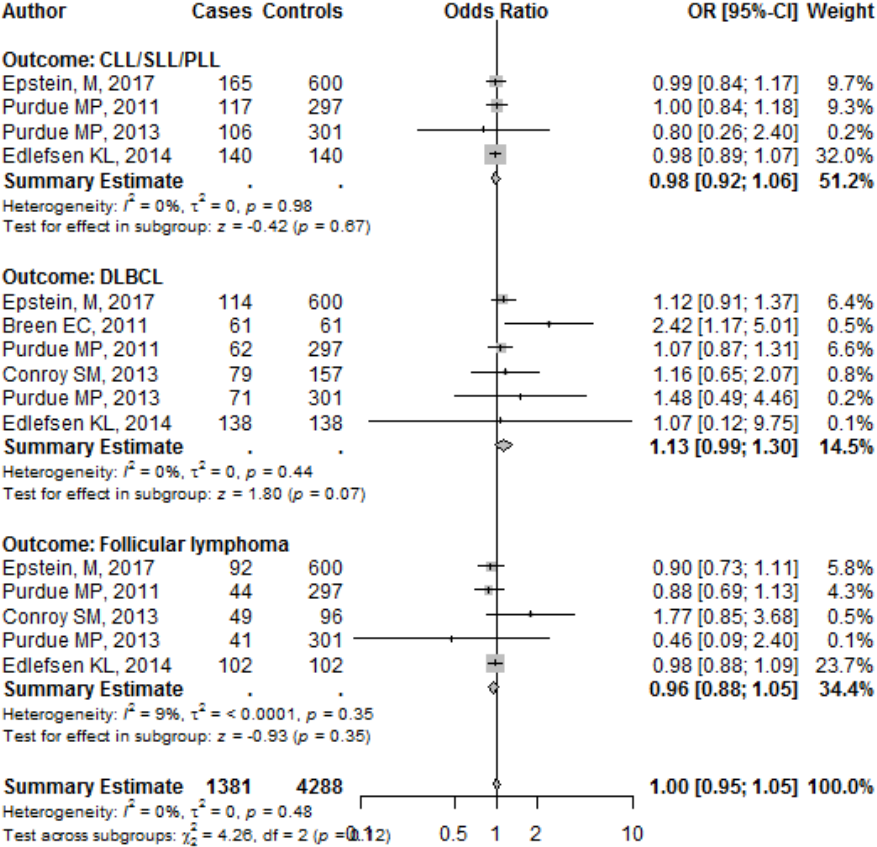


**IL-6**


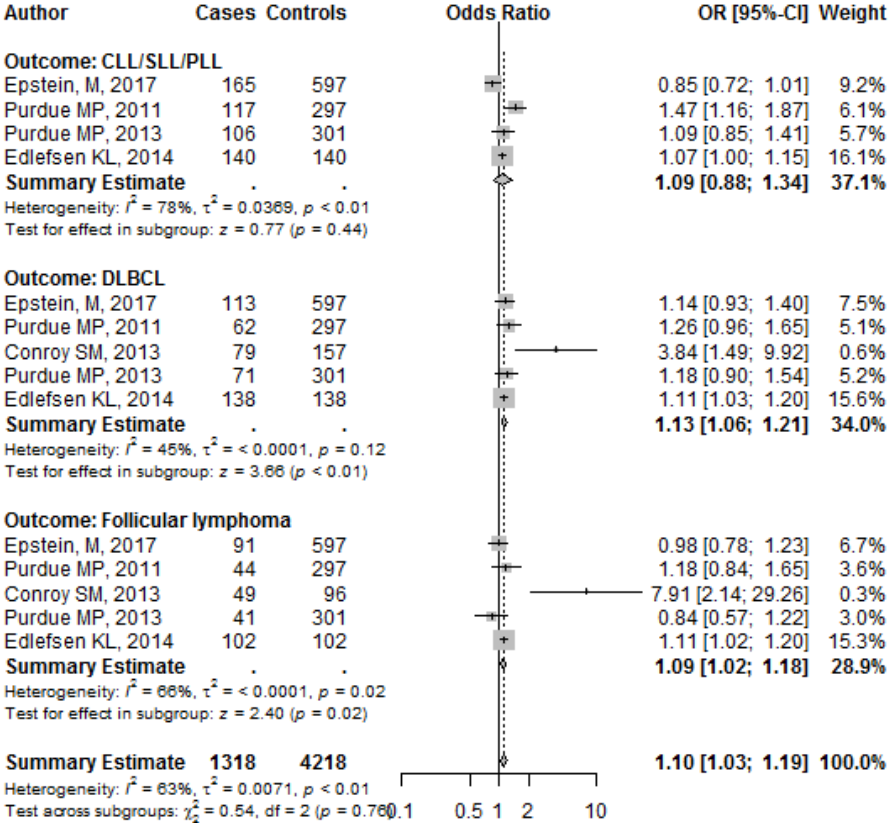


**IL-10**


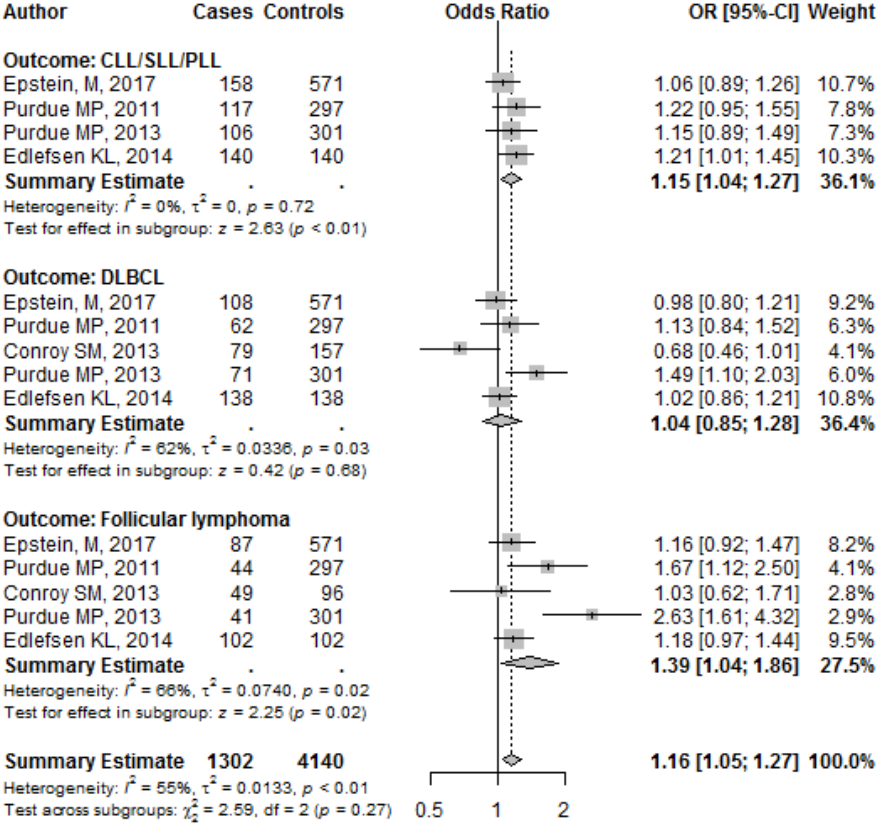


**TNF-α**


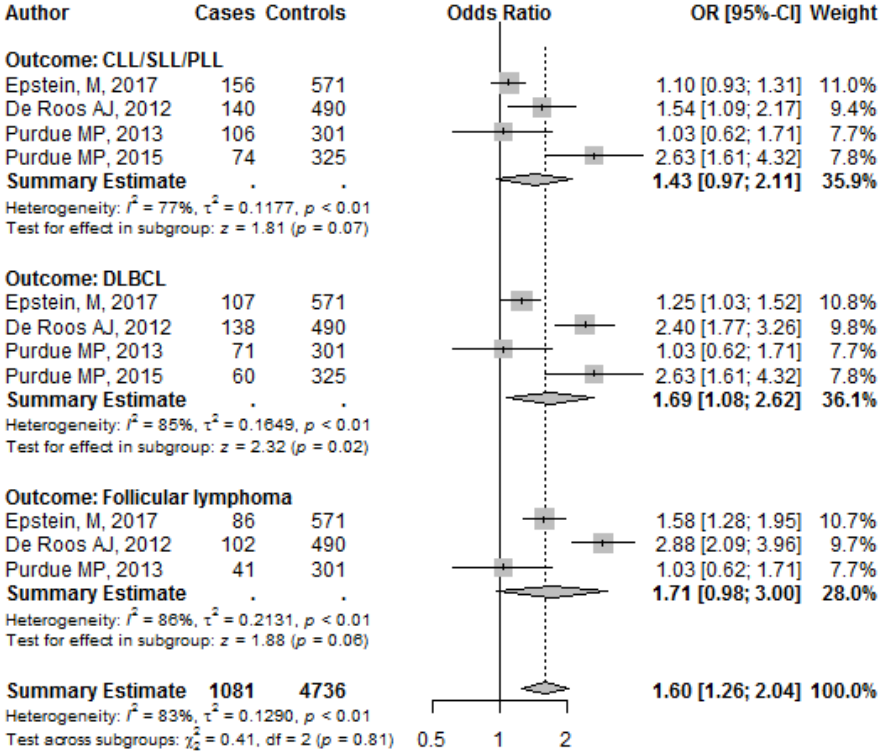


**CXCL13**


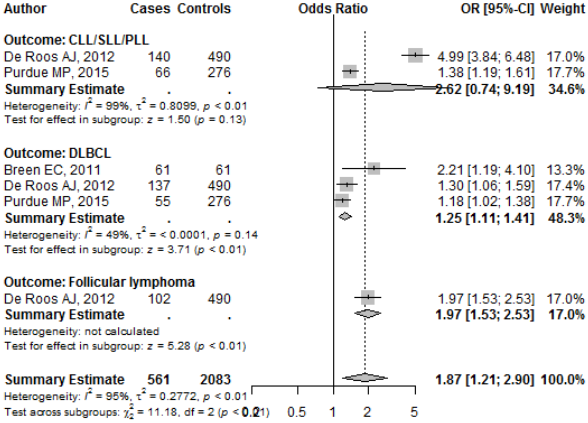


**sCD23**

^22^


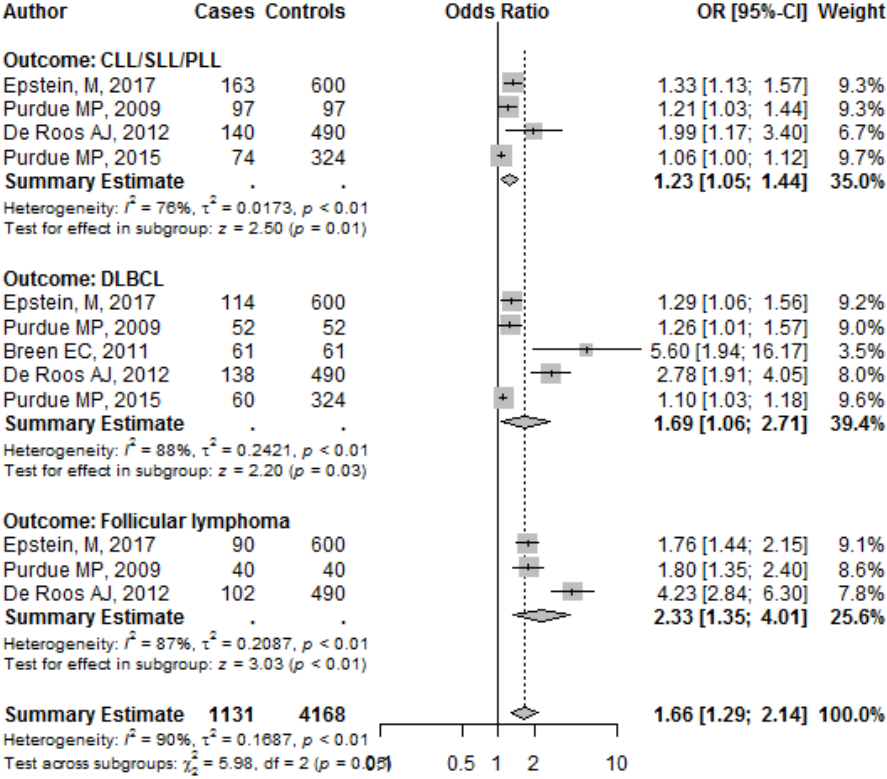


**sCD30**

**Figure S1 Legend:** Error bars indicate 95% CIs; size of the squares indicates the precision weight of each study in the random-effects meta-analysis. Diamonds indicate the summary ORs. Case and control sample sizes for were estimated for Purdue, 2009^22^.

**Supplementary Figure S2:** Funnel Plots Assessing Publication Bias


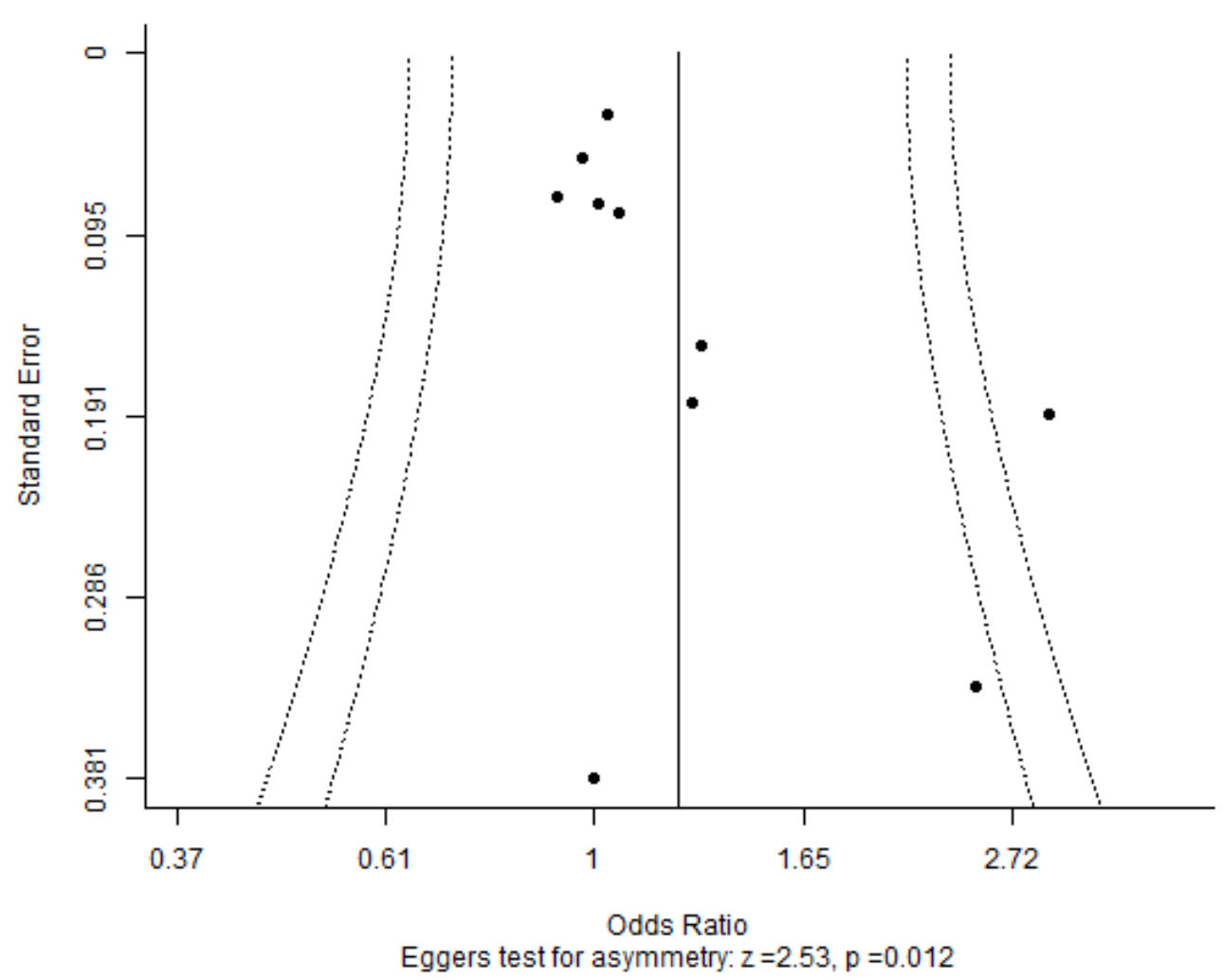


**IL-6**


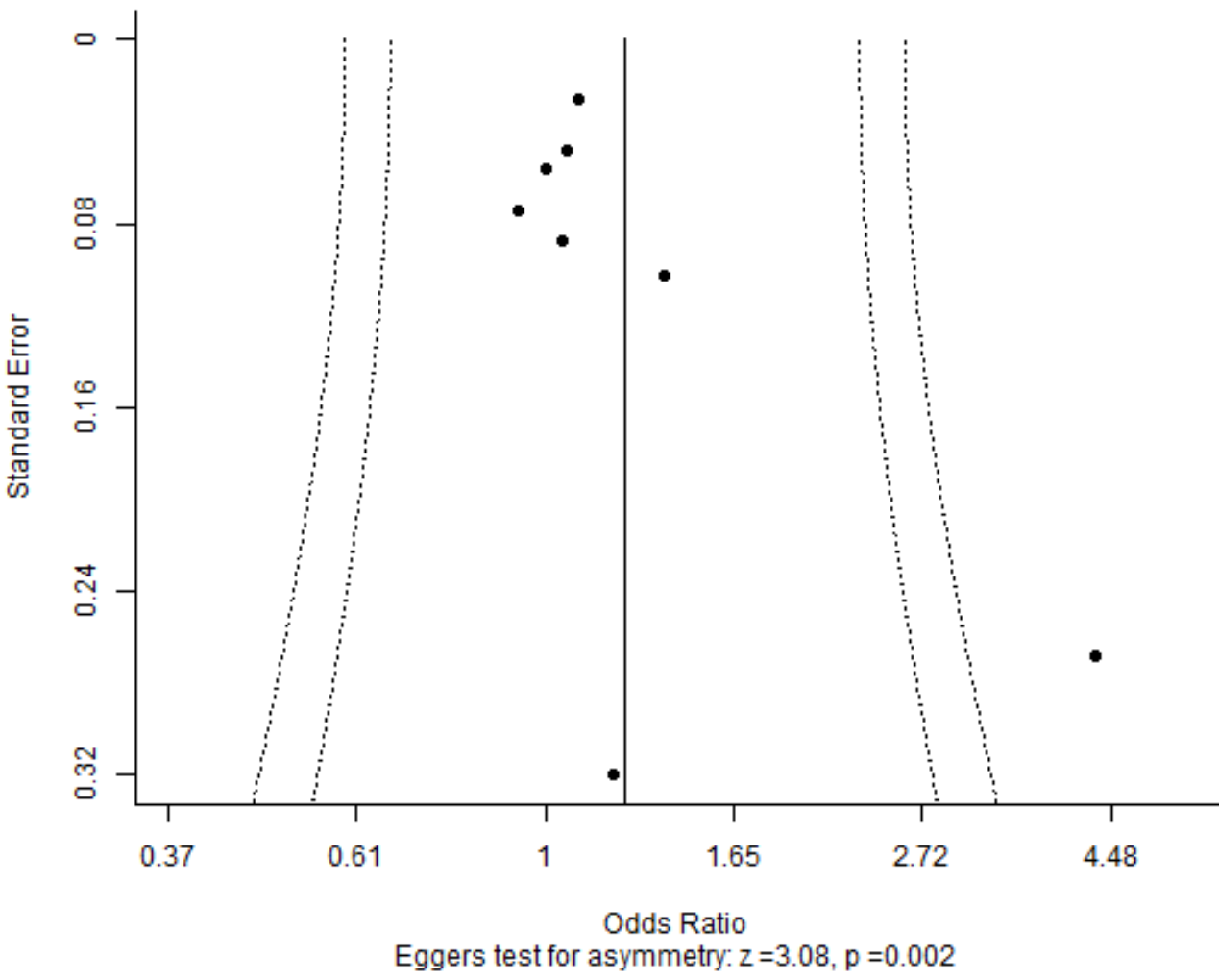


**IL-10**


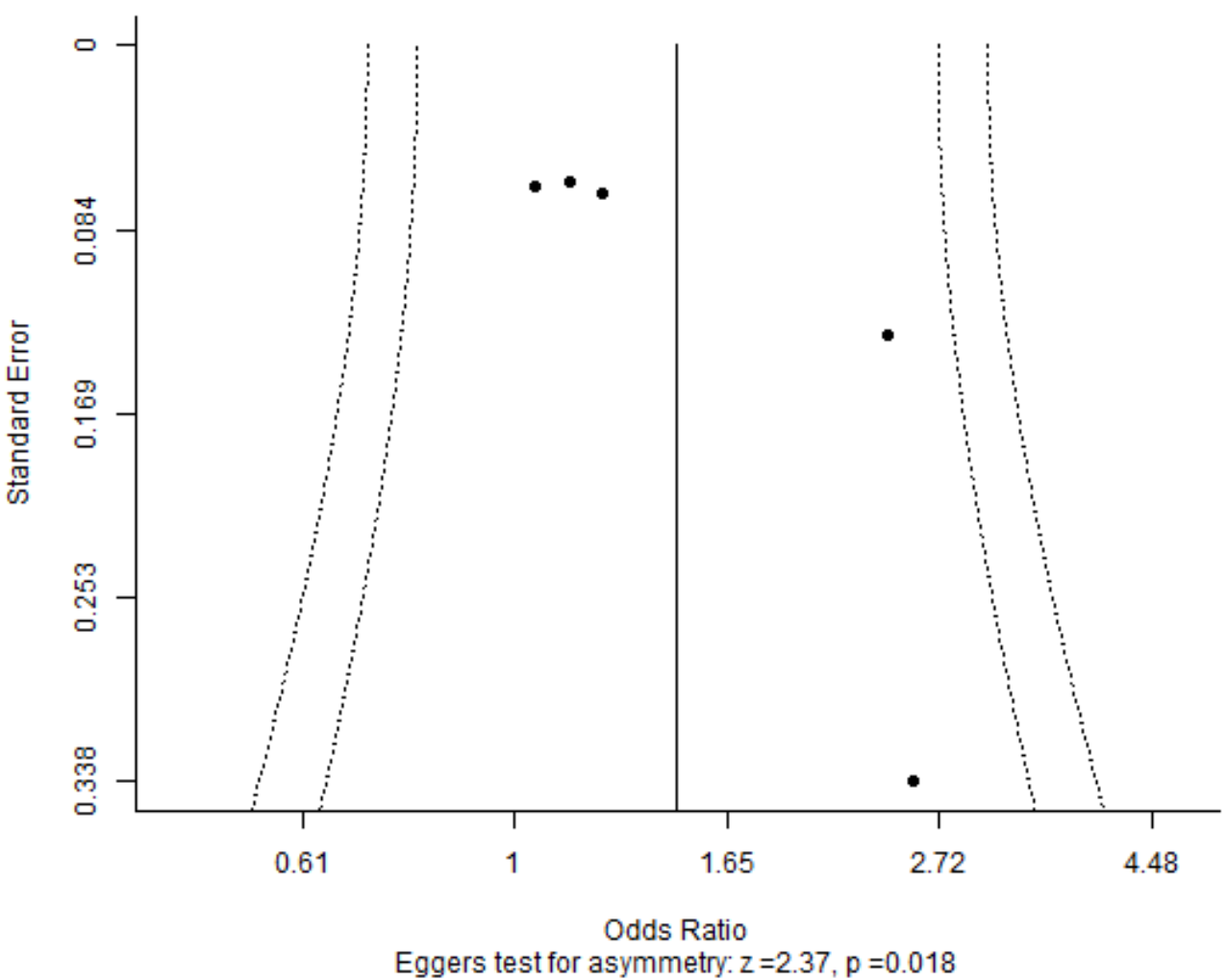


**CXCL13**


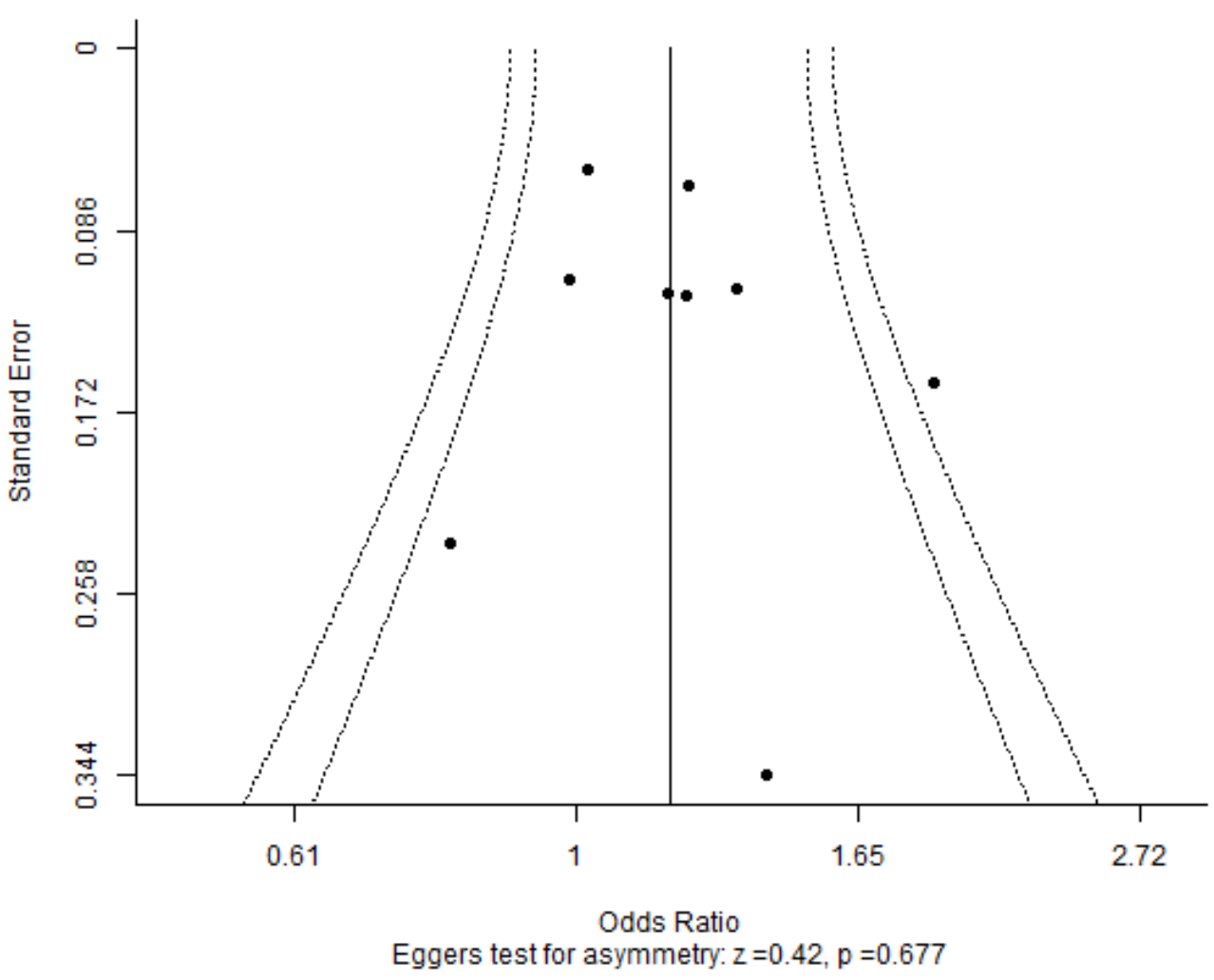


**TNF-α**


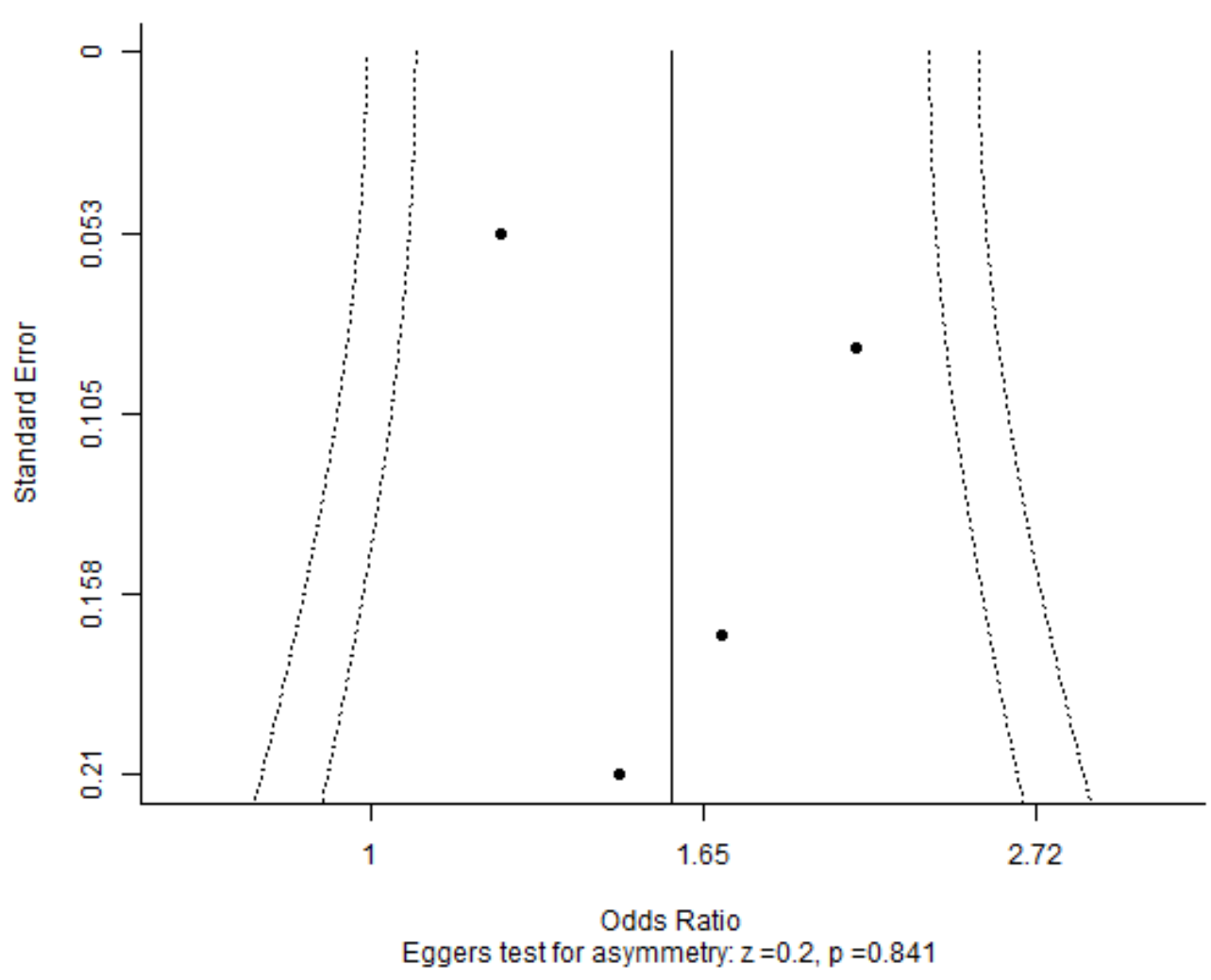


**sCD23**


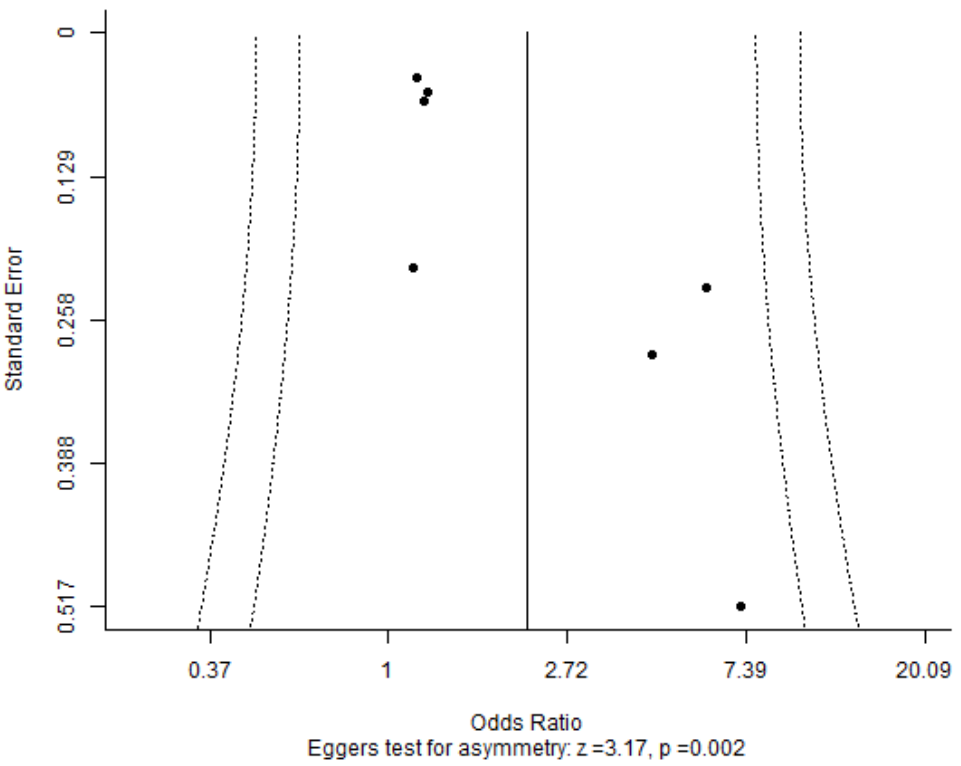


**sCD27**


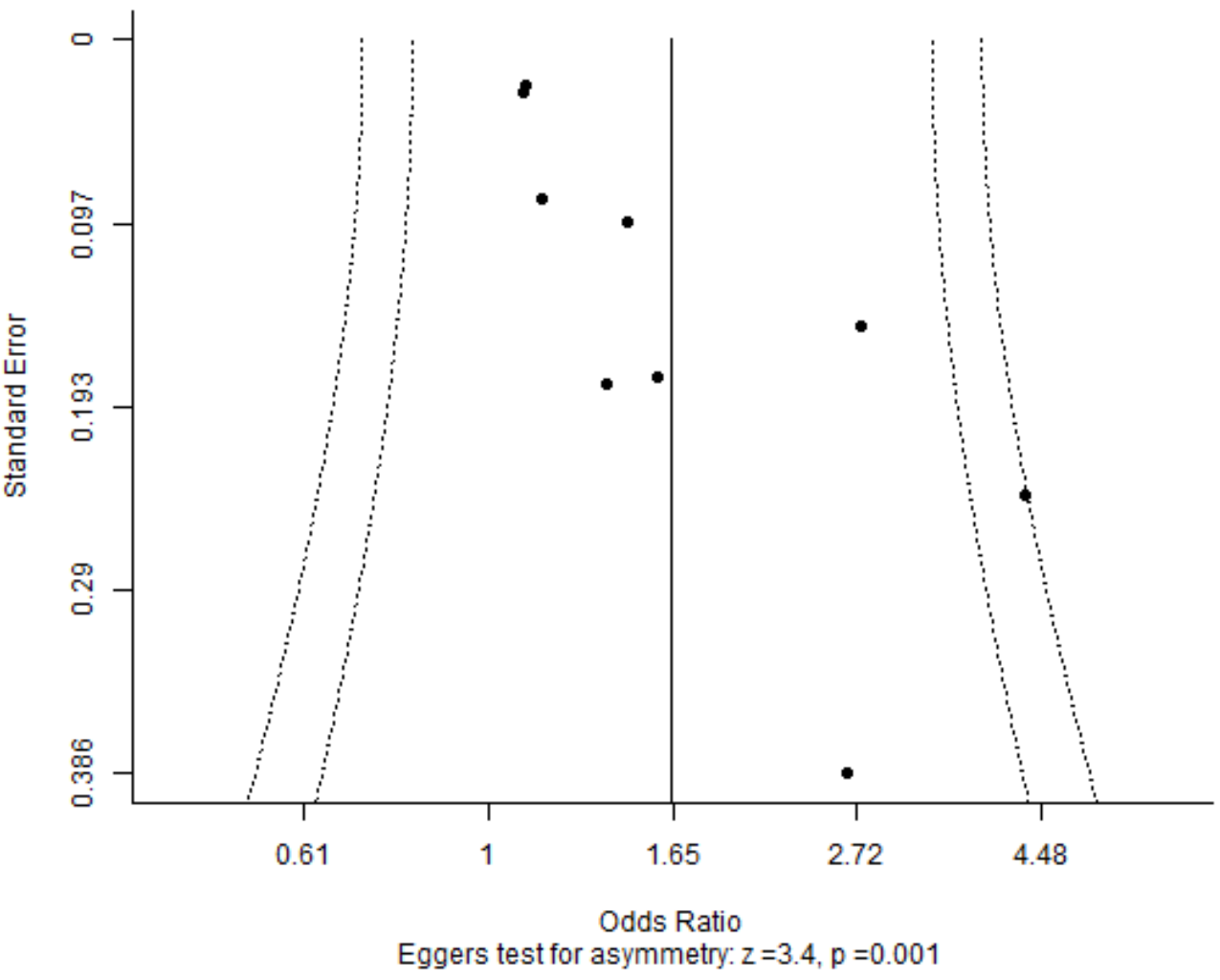


**sCD30**

**Figure S2 Legend:** Funnels indicate pseudo 90% (outer) and 95% (inner) confidence intervals accounting for between study heterogeneity estimated from the random effects models. Each plot assesses the potential influence of publication bias on the association between a given biomarker and the overall NHL outcome. Vertical reference lines drawn at point estimate for each analysis. Analyses and assessments of publication bias are not independent since most publications examined multiple biomarkers.

**Supplementary Table S2:** Bias Analyses: Egger's Regression P-values and Trim & Fill Analyses

|  | **Summary of** | |  |  | | | |
| --- | --- | --- | --- | --- | --- | --- | --- |
|  | **Observed Data** | |  |  |  | **After Trim-and-Fill ^*^** | |
| **Biomarker** | **N** | **OR 95% CI** |  | **Egger's P** |  | **N** | **OR 95% CI** |
| IL-6 | 10 | 1.22 [0.97, 1.54] |  | 0.012 |  | 13 | 1.02 [0.76, 1.37] |
| IL-10 | 8 | 1.24 [0.93, 1.63] |  | 0.002 |  | 9 | 1.09 [0.72, 1.65] |
| TNF-α | 9 | 1.18 [1.04, 1.34] |  | 0.677 |  | 10 | 1.12 [0.95, 1.33] |
| sCXCL13 | 5 | 1.47 [1.03, 2.08] |  | 0.018 |  | 6 | 1.32 [0.89, 1.94] |
| sCD23 | 4 | 1.57 [1.21, 2.05] |  | 0.841 |  | 6 | 1.25 [0.90, 1.74] |
| sCD27 | 7 | 2.18 [1.20, 3.98] |  | 0.002 |  | 9 | 1.39 [0.63, 3.06] |
| sCD30 | 9 | 1.65 [1.22, 2.22] |  | <0.001 |  | 14 | 1.13 [0.78, 1.64] |
| * Trim-and-fill analyses impute data from studies predicted to have possibly been excluded due to publication bias and re-estimate the summary estimates. OR represent the summary estimates including these hypothetically missing studies. | | | | | | | |

**Supplementary Figure S3:** Influence Diagnostics: Leave-One-Out Analyses


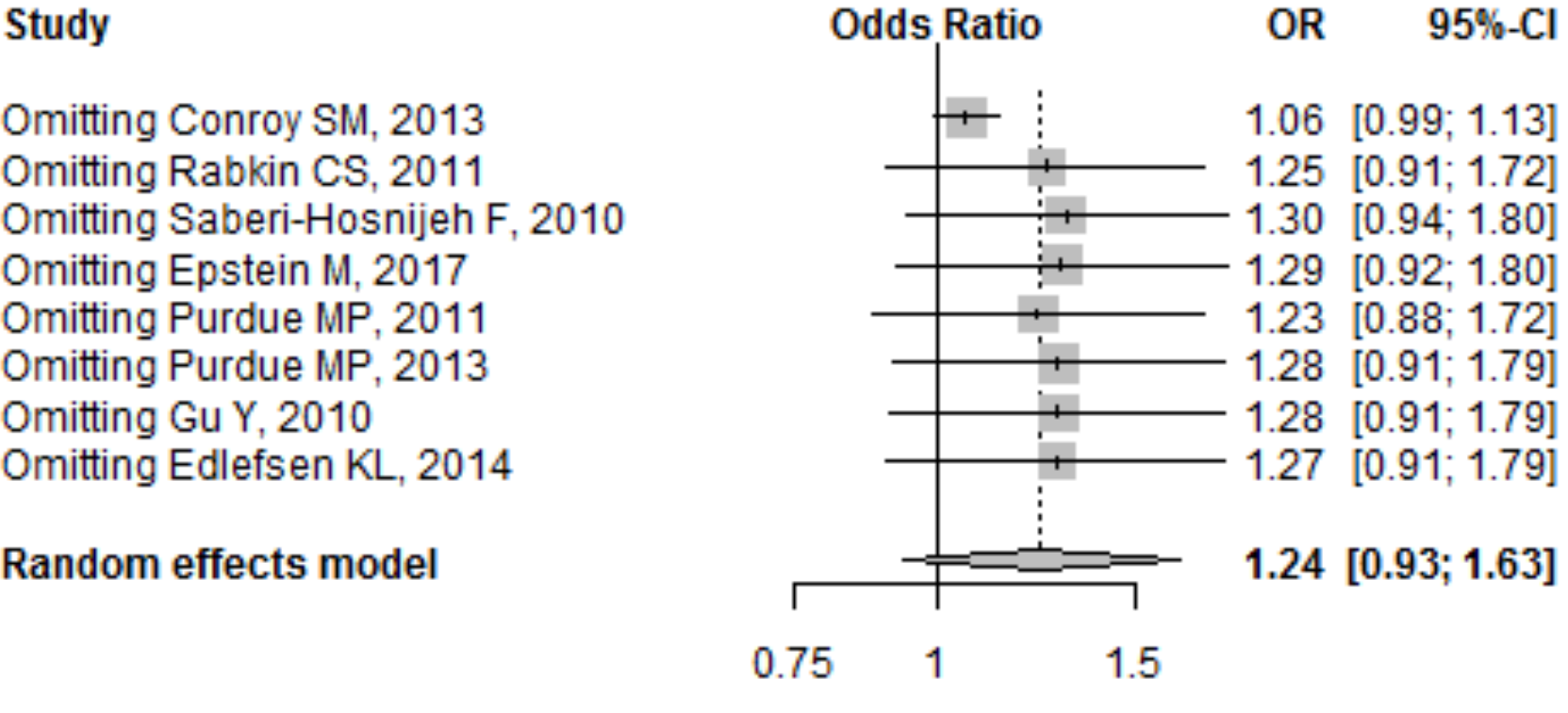


**IL-10**


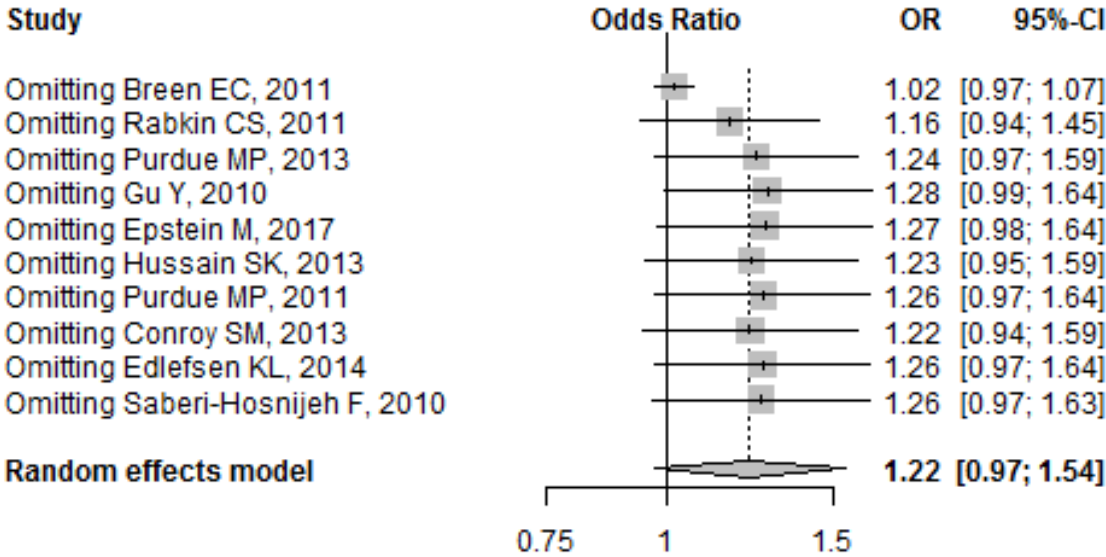


**IL-6**


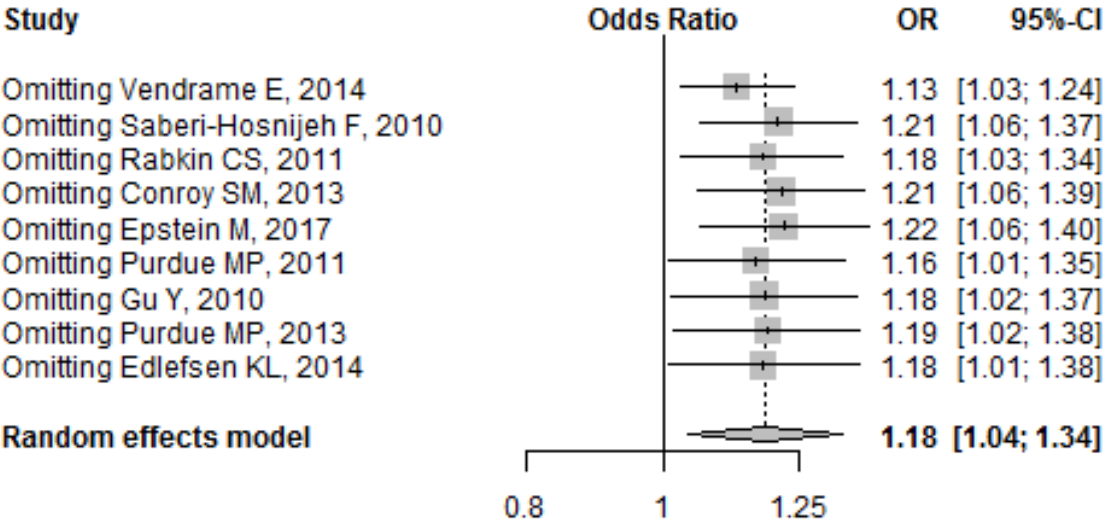


**TNF-α**


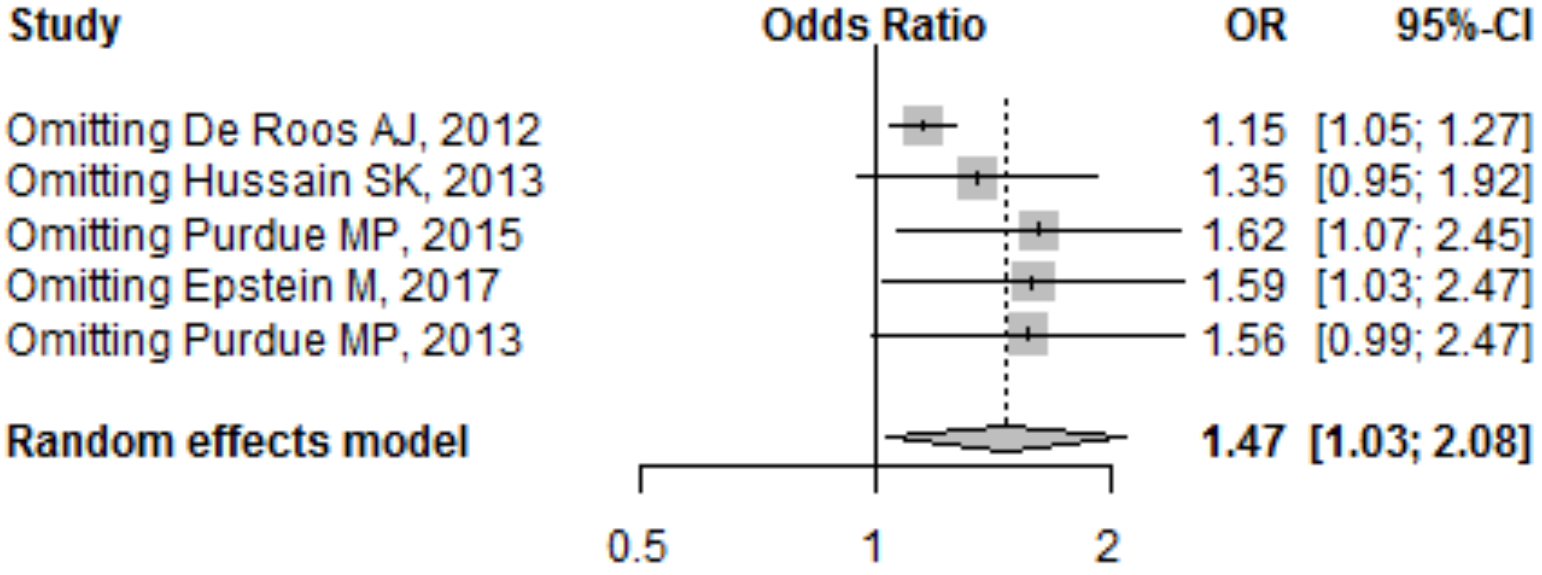


**CXCL13**


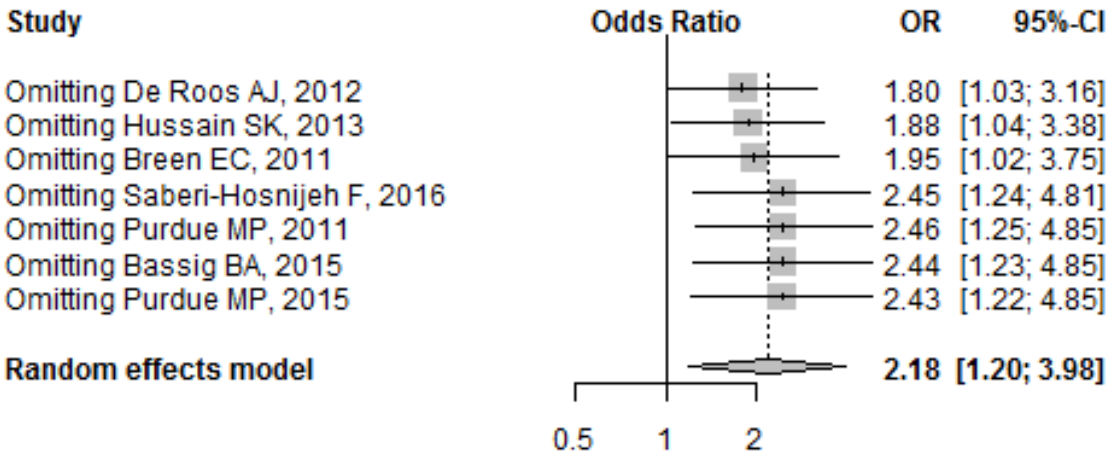

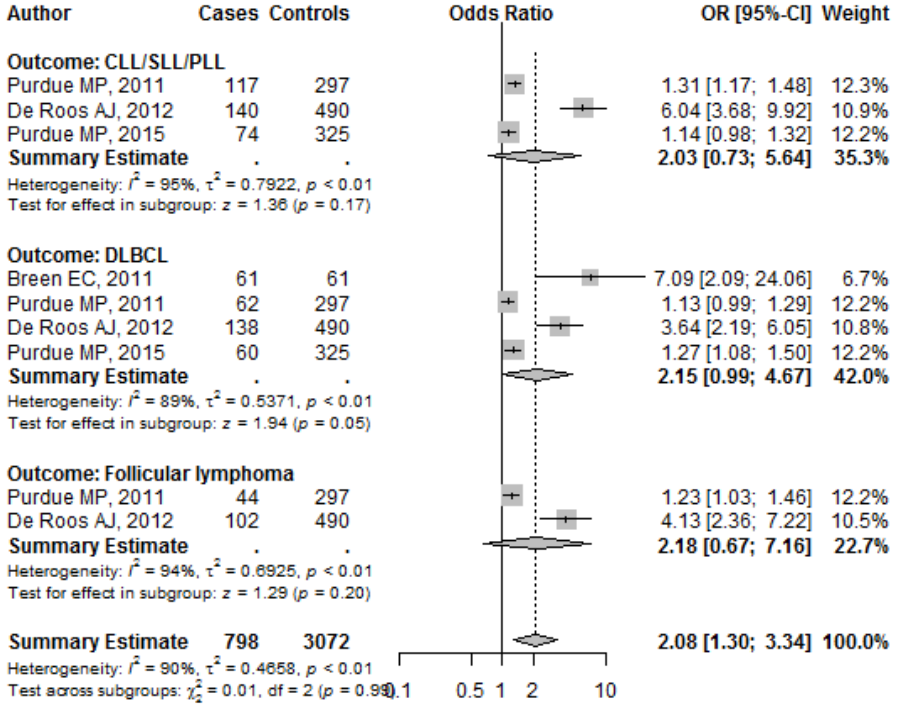


**sCD27ssCD27**


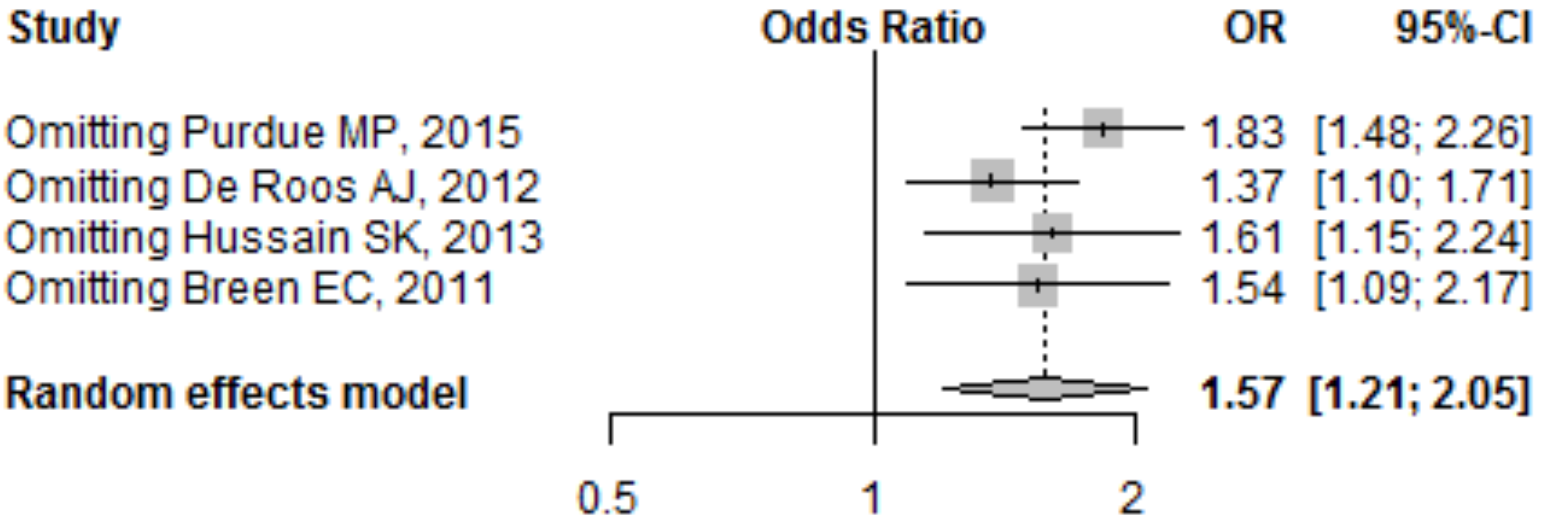


**sCD23ssCD23**


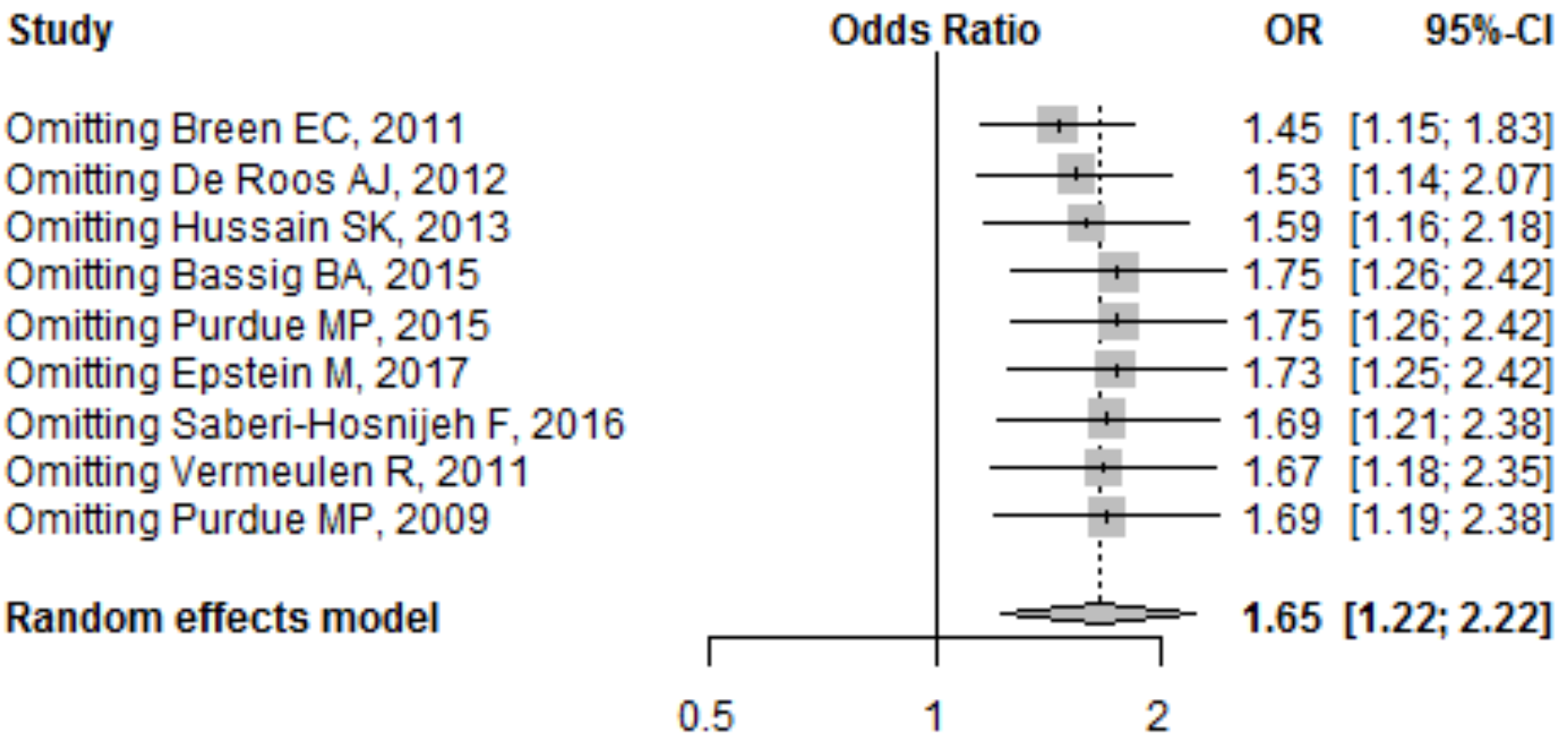


**sCD30**

**Figure S3 Legend:** Leave-one-out diagnostic analyses showing the effect of removing a given study on the summary meta-analytic estimate. Random effects model refers to the summary OR estimate calculated in meta-analyses.

**Supplementary Table S3:** Results for All B-Cell NHL among HIV-infected: Comparing HAART exposed versus Unexposed

|  |  | **HAART Exposure Status** | | | | | |  |
| --- | --- | --- | --- | --- | --- | --- | --- | --- |
|  |  |  |  |  |  |  | **Meta-Regression** |  |
|  |  | **Unexposed*** |  |  | **Exposed*** |  | **Comparison of** |  |
| **Analyte** | **N** | **OR 95% CI** | **I^2^** | **N** | **OR 95% CI** | **I^2^** | **Unexposed vs. Exposed** | **P** |
| IL-6 **‡** | 3 | 2.08 [1.17, 3.70] | 75 [92, 18] | 1 | 1.37 [0.75, 2.52] | 0 [-,-] | 1.52 [0.46, 5.01] | 0.493 |
| sCD23 | 2 | 1.75 [1.30, 2.36] | 0 [-,-] | 1 | 1.18 [0.61, 2.30] | 0 [-,-] | 1.48 [0.72, 3.08] | 0.288 |
| sCD27 | 2 | 4.72 [2.81, 7.93] | 0 [-,-] | 1 | 13.07 [1.87, 91.52] **†** | 0 [-,-] | 0.36 [0.05, 2.71] | 0.322 |
| sCD30 | 2 | 4.14 [2.71, 6.32] | 11 [-,-] | 1 | 1.55 [0.46, 5.26] | 0 [-,-] | 2.67 [0.73, 9.73] | 0.137 |

**Notes:**

* The unexposed group comprises studies comprised 1 study including HAART naive participants. The other 2 studies with HAART unexposed participants adjusted for HAART, implying the estimate presented is among the reference group of HAART unexposed individuals, which we include in these analyses (4,15,16).

† In the publication assessing HAART exposed individuals (5), there were 9 HAART exposed cases and 37 controls, which led to an inflated estimate.

‡ These analyses included Vendrame, 2014 and Breen, 2011 which contain completely overlapping study subjects, but different assay technologies. We include them here, but not in the manuscript because the results are not substantially different with or without exclusion, and given the small sample size, the additional information dominates the small bias due to lack of independence for our assessment of HAART exposure associations and their differences. Analyses presented only if at least one stratum had 2 or more studies.

**Supplementary Table S4:** Multiple Comparisons Adjusted P-values for Table 2

| **Multiple Comparisons** |  | **P-Value Adjustment** | | |
| --- | --- | --- | --- | --- |
| **Group** | **Biomarker** | **Unadjusted** | **Stepdown Bonferroni*** | **Dependent FDR*** |
| Overall | CXCL13 | 0.0320 | 0.0959 | 0.1160 |
|  | IL-10 | 0.1375 | 0.1651 | 0.3564 |
|  | IL-6 | 0.0825 | 0.1651 | 0.2497 |
|  | TNF-α | 0.0100 | 0.0498 | 0.0498 |
|  | CD23 | 0.0008 | 0.0055 | 0.0102 |
|  | CD27 | 0.0110 | 0.0498 | 0.0498 |
|  | CD30 | 0.0011 | 0.0068 | 0.0102 |
| HIV- | CXCL13 | 0.0944 | 0.4429 | 0.4265 |
|  | IL-10 | 0.1741 | 0.4429 | 0.5267 |
|  | IL-6 | 0.6025 | 0.6025 | 1.0000 |
|  | TNF-α | 0.0151 | 0.0908 | 0.1374 |
|  | CD23 | 0.0886 | 0.4429 | 0.4265 |
|  | CD27 | 0.1175 | 0.4429 | 0.4265 |
|  | CD30 | 0.0043 | 0.0298 | 0.0772 |
| HIV+ | CXCL13 | 0.0054 | 0.0161 | 0.0195 |
|  | IL-10 | 0.5683 | 0.5683 | 1.0000 |
|  | IL-6 | 0.0101 | 0.0201 | 0.0304 |
|  | TNF-α | <.0001 | 0.0003 | 0.0003 |
|  | CD23 | 0.0004 | 0.0017 | 0.0019 |
|  | CD27 | <.0001 | <.0001 | <.0001 |
|  | CD30 | <.0001 | <.0001 | <.0001 |
| Meta-Regression | CXCL13 | 0.2181 | 0.6543 | 0.7917 |
|  | IL-10 | 0.9428 | 1.0000 | 1.0000 |
|  | IL-6 | <.0001 | <.0001 | <.0001 |
|  | TNF-α | 0.0052 | 0.0262 | 0.0317 |
|  | CD23 | 0.9959 | 1.0000 | 1.0000 |
|  | CD27 | 0.0413 | 0.1651 | 0.1873 |
|  | CD30 | 0.0029 | 0.0177 | 0.0268 |

**Notes:**

*Methods used described in Holm, 1979 and Benjamini & Yekutieli, 2001, were deemed appropriate for tests that may contain statistical dependence since biomarker biological functions are not independent.
